# Extracellular matrix and perineuronal nets in neural plasticity, memory, and epileptogenesis

**DOI:** 10.3389/fnmol.2026.1945520

**Published:** 2026-09-15

**Authors:** Hanxiao Zhu, Qiongfen Li, Yun Li, Wei Wang

**Affiliations:** 1 Department of Neurology, The First Affiliated Hospital of Dali University, Clinical Medical School, Health Science Center, Dali University, Dali, China; 2 College of Basic Medical, Health Science Center, Dali University, Dali, China

**Keywords:** chondroitin sulfate proteoglycans, epilepsy, extracellular matrix, hyaluronan, perineuronal nets, synaptic plasticity

## Abstract

In the mature brain, the extracellular matrix (ECM) molecules derived from neurons and glia accumulate in spatially distinct extracellular compartments, including a diffuse interstitial matrix and condensed perineuronal nets (PNNs), where they regulate receptor mobility, ion homeostasis, cell-surface signaling, structural stabilization, and experience-dependent synaptic remodeling. PNNs, in particular, assemble around selected neuronal populations - most prominently parvalbumin-positive fast-spiking interneurons - and thereby couple extracellular molecular architecture to inhibitory control, critical-period closure, and the long-term precision of network activity. These properties place ECM and PNNs at the intersection of developmental maturation, synaptic plasticity, memory-related stabilization, and pathological circuit remodeling. These principles are directly relevant to epilepsy. If ECM and PNNs normally stabilize synaptic organization, preserve inhibitory interneuron function, regulate extracellular space, and constrain maladaptive rewiring, then their disruption is positioned to lower the threshold for persistent hyperexcitability and aberrant network synchronization. For instance, hyaluronan deficiency alters neuronal activity and can produce seizures. However, the biological mechanisms and specific contributions of ECM and PNN in epilepsy still need to be further elucidated. Therefore, the purpose of the study is to systematically elaborate on the similarities and differences between ECM and PNN in terms of plasticity, memory, the biological mechanisms of neurological diseases, especially in the context of epilepsy.

## 1 Introduction

The conceptual status of the brain ECM has changed profoundly over the past two decades (Dityatev and Schachner, 2003; Wlodarczyk et al., 2011; Lugara et al., 2026). For many years, it was treated largely as inert extracellular material or as a developmental residue whose major importance lay outside mature circuit physiology (useful for structural support). However, that view is no longer defensible. The recent studies shows that the neural ECM is a molecularly heterogeneous, spatially organized, and dynamically remodeled system that participates directly in neuronal development, synaptic regulation, memory-related remodeling, injury responses, and repair (Melrose et al., 2021; Fawcett et al., 2022; Li et al., 2025). In the adult CNS, extracellular molecules are not merely present between cells; they form functional microenvironments that shape how neurons signal, stabilize contacts, and respond to experience (Dityatev and Schachner, 2003; Melrose et al., 2021; Fawcett et al., 2022). In this sense, the extracellular compartment belongs to the causal machinery of neural computation rather than to its anatomical background.

This revised perspective is especially important because the CNS ECM is fundamentally unlike the fibrillar, collagen-dominated matrices of classical connective tissues. The mature brain contains a highly hydrated and chemically specialized ECM enriched in proteoglycans, glycosaminoglycans, and glycoproteins that regulate tissue homeostasis, development, and regeneration despite the relative paucity of conventional stabilizing collagenous and elastic structures (Li et al., 2025; Kwok and Dityatev, 2026). Among these components, chondroitin sulfate proteoglycans (CSPGs), heparan sulfate proteoglycans (HSPGs), hyaluronan, tenascins, and associated link proteins form a molecular system that is at once structural and instructive (Galtrey and Fawcett, 2007; Galtrey et al., 2008). It can bind guidance cues, shape extracellular diffusion, influence membrane receptor organization, and confer cell-directive properties on the extracellular space (Melrose et al., 2021; Kwok and Dityatev, 2026). Thus, rather than forming an inert filler between cells, the ECM provides a molecular interface through which neurons, glia, and the vasculature regulate each other over multiple spatial and temporal scales. This is particularly evident in specialized ECM compartments such as the peri-synaptic matrix, the perinodal ECM, and the PNN, each of which imposes local rules on circuit function (Wang and Fawcett, 2012).

The ECM exists in distinct anatomical forms. One component is the diffuse interstitial matrix that permeates the neuropil and surrounds synapses throughout the CNS (Syková and Nicholson, 2008). Superimposed on this is a more condensed, lattice-like specialization: the perineuronal net (Auer et al., 2025). PNNs enwrap the soma and proximal dendrites of selected neuronal populations, particularly inhibitory parvalbumin-positive (PV) interneurons, and are now viewed as one of the key extracellular correlates of circuit maturation (Härtig et al., 1994; Alzarea, 2026). Their emergence during postnatal development coincides with the restriction of juvenile-like plasticity, yet their function is not simply suppressive. Rather, their organization around the soma and proximal dendrites of metabolically demanding, high-frequency neurons suggests that they also buffer ions, regulate molecular diffusion, protect against oxidative or excitotoxic stress, and preserve the temporal precision of mature circuits (Soleman et al., 2013; Fawcett et al., 2022). Therefore, PNNs help convert an initially permissive and labile developmental environment into one in which plasticity persists in a more selective, stable, and energetically constrained form (Celio et al., 1998; Carulli et al., 2010; Fawcett et al., 2022).

In brain, different ECM compartments, different matrix molecules, and different sulfation states can exert sharply different biological effects depending on cell type, developmental stage, and physiological context. In some settings, ECM attenuation reopens plasticity (Frischknecht et al., 2009; Singh et al., 2026). In others, loss of defined extracellular components impairs long-term potentiation, alters inhibitory circuitry, or destabilizes established synaptic architecture (Dzyubenko and Hermann, 2025; Nakajo et al., 2026). Therefore, future study is not on qualitatively whether the ECM promotes or suppresses plasticity, but how extracellular organization determines where plasticity may occur, what threshold it requires, and whether the resulting change is transient, consolidated, or maladaptive.

Interestingly, extracellular regulation in the brain is not neuron-exclusive. ECM molecules are produced by both neurons and glia, and their remodeling reflects multicellular coordination rather than the passive aftermath of neuronal firing (Dityatev and Schachner, 2003; Hodebourg et al., 2025). Astrocytes contribute to the organization of the perisynaptic extracellular environment, while microglia actively remodel extracellular material in ways that affect synapse plasticity (Nguyen et al., 2020; Dzyubenko and Hermann, 2025; Hodebourg et al., 2025). This means that the ECM is best understood as an intercellular interface through which neurons, glia, and extracellular effectors cooperatively shape circuit state. Mature plasticity, in other words, is partly an extracellular systems property.

These characteristics may make ECM biology especially relevant to epilepsy (Lugara et al., 2026). Epilepsy are not merely conditions of transient hypersynchronous discharges; they are disorders of circuit stability, extracellular homeostasis, and activity-dependent remodeling. If ECM and PNNs normally stabilize synapses, preserve inhibitory interneuron function, regulate extracellular volume, and constrain aberrant structural change, then disruption of these extracellular systems is plausibly positioned to facilitate epileptogenesis. The observation that hyaluronan deficiency can alter neuronal activity and precipitate seizures is especially revealing because it links a fundamental matrix component to network excitability in a direct mechanistic way (Arranz et al., 2014; Perkins et al., 2017). In this light, epilepsy should not be considered separately from ECM biology (an accessory phenomenon), but as one of the pathological contexts in which the balance between extracellular stabilization and plastic remodeling may fail (Arranz et al., 2014; Fawcett, 2015; Fawcett et al., 2022). The implications of this mechanism for epilepsy are discussed later in Section 6; they are not repeated there in full.

Based on this, the purpose of the study is to provide a systematic account of ECM and PNN biology across plasticity, memory, neurological disease, and epilepsy. First, the molecular organization and developmental emergence of the ECM and PNNs are considered. Second, their roles in synapses and plasticity are analyzed. Third, their contribution to memory and neurological disorders is summarized. Finally, our study addresses epilepsy in detail, with particular focus on matrix metalloproteinase-9 (MMP-9), seizure-induced PNN disruption, inflammatory and blood–brain barrier mechanisms, and the therapeutic problem of whether extracellular remodeling should be restrained, redirected, or transiently reopened.

## 2 Structure, distribution, and ontogeny of the ECM and PNNs

### 2.1 Diffuse/interstitial and perisynaptic ECM

The ECM of the CNS is a highly specialized molecular system whose organization differs fundamentally from that of connective tissues (Melrose et al., 2021; Kwok and Dityatev, 2026). In most peripheral organs, extracellular architecture is dominated by fibrillar collagens and basement membrane assemblies that provide tensile strength and structural support (Mienaltowski et al., 2021). By contrast, the neural ECM is comparatively poor in fibrillar collagen and is instead composed predominantly of hydrated glycosaminoglycans, proteoglycans, link proteins, and glycoproteins, such as hyaluronan (HA) and CSPGs (Dityatev and Schachner, 2003; Deepa et al., 2006; Oohashi et al., 2015). This distinction is not merely biochemical; it reflects the unique physical demands of nervous tissue, where synaptic communication, extracellular diffusion, ionic buffering, and activity-dependent structural remodeling must be accommodated within a densely packed and electrically excitable milieu. The brain ECM is therefore not an inert filler occupying intercellular gaps. It is a molecular infrastructure that shapes extracellular space geometry, regulates diffusion of neurotransmitters and ions, presents ligands to cell-surface receptors, and contributes directly to neuronal excitability and network behavior (Fawcett et al., 2022; Hodebourg et al., 2025).

At a broad level, one can distinguish the diffuse interstitial matrix, the perisynaptic ECM, the condensed PNN surrounding neuronal somata and proximal dendrites, and the perinodal ECM associated with nodes of Ranvier (Fawcett et al., 2019). These compartments are continuous in principle but molecularly differentiated in practice, and this heterogeneity is essential to their function. At the molecular level, the central organizing scaffold of the neural ECM is HA, a long, non-sulfated glycosaminoglycan synthesized at the plasma membrane by hyaluronan synthases (HAS) (Galtrey and Fawcett, 2007; Galtrey et al., 2008). In the CNS, HA is not simply a hydrated polymer thatupies space; rather, it provides the backbone on which major matrix macromolecules are assembled. Among these, the lectican family of CSPGs-aggrecan, brevican, neurocan, and versican-occupies a privileged position. Their N-terminal globular domains bind HA, whereas the interaction is stabilized by hyaluronan and proteoglycan link proteins (HAPLNs) (Yamaguchi, 2000; Wang and Fawcett, 2012). Their C-terminal domains, in turn, bind glycoproteins such as tenascin-R (TnR), thereby allowing formation of higher-order supramolecular lattices. Yamaguchi proposed this organization as the “HLT” model, emphasizing the core structural triad of hyaluronan, lecticans, and tenascin-R, and this conceptual framework remains central to current understanding of PNN assembly (Aspberg et al., 1997; Yamaguchi, 2000; Oohashi et al., 2015). In addition, other matrix-associated molecules-including phosphacan/RPTPζ, heparan sulfate proteoglycans, thrombospondins, and tenascin-C-broaden the signaling and binding repertoire of the CNS ECM.

This molecular composition gives the neural ECM several functional properties of direct physiological relevance. By regulating the geometry and hydration of extracellular space, HA-rich matrix influences the diffusion of ions, metabolites, and transmitter molecules. It also contributes to the microenvironment in which receptors, adhesion molecules, and voltage-sensitive membrane proteins operate (Li et al., 2025; Kwok and Dityatev, 2026). Importantly, the functional behavior of CSPGs is not determined solely by the identity of the core proteins. Their glycosaminoglycan chains carry sulfation motifs that profoundly modify molecular interactions. In the adult CNS, 4-sulfated chondroitin sulfate motifs are generally more inhibitory, whereas 6-sulfated motifs are relatively permissive for axon growth and plasticity (Fawcett and Kwok, 2022). Thus, the matrix is not only structurally organized but chemically encoded. As a result, neural ECM exhibits strong fixed negative charge, high water-binding capacity, cation-buffering properties, and remarkable ability to sequester growth factors, guidance molecules, and extracellular modulators (Yamaguchi, 2000; Oohashi et al., 2015; Dzyubenko and Hermann, 2025; Hodebourg et al., 2025). Meanwhile, the neural ECM should be viewed as a composite informational material, in which supramolecular architecture and sulfation chemistry jointly determine permissiveness, ligand binding, and stability (Miyata et al., 2012; Kwok and Dityatev, 2026).

### 2.2 PNN architecture and cellular distribution

Notably, the brain ECM is not a homogeneous gel. Rather, its components self-organize into anatomically and functionally distinct extracellular compartments (Fawcett et al., 2019). One form is the diffuse or interstitial ECM, which permeates the neuropil and surrounds essentially all cellular elements. Another is the PNN, a condensed reticular specialization that enwraps neuronal somata and proximal dendrites (Sterin et al., 2024; Kwok and Dityatev, 2026). These are not biochemically unrelated entities. They are built from overlapping molecular repertoires, but differ in local concentration, cross-linking density, ultrastructural condensation, and topological relation to neuronal membranes and synaptic boutons. PNNs are therefore best regarded not as a separate matrix class, but as a highly ordered state of ECM assembly. Their lattice-like morphology emerges because HA-bound CSPGs are stabilized by HAPLNs and cross-linked by TnR into a condensed extracellular coat that nonetheless preserves discontinuities at sites of synaptic contact (Celio et al., 1998; Carulli et al., 2010; Morawski et al., 2014). This architecture explains a fundamental paradox of PNN biology: they stabilize local neuronal microenvironments without sealing neurons off from synaptic input (Fawcett et al., 2022).

Although aggrecan is widely considered the canonical and often indispensable PNN component, mature nets contain multiple CSPGs in region-specific and cell-type-specific combinations, including brevican, neurocan, versican, and phosphacan (Yamaguchi, 2000; Wang and Fawcett, 2012; Singh et al., 2026). The relative abundance of these constituents differs across cortex, hippocampus, cerebellum, brainstem, and spinal cord. Moreover, even among nets built around similar neuronal classes, there are variations in HAPLN usage, *Wisteria floribunda* agglutinin reactivity, and the association with additional ligands such as semaphorin 3A (Carulli et al., 2006; Oohashi et al., 2015). Some nets are WFA-rich and tightly condensed, whereas others are compositionally broader, less intensely labeled, or associated with distinct synaptic arrangements. Such heterogeneity is unlikely to be merely decorative. Different combinations of lecticans and glycoproteins almost certainly confer different biomechanical properties, ligand-binding capacities, and capacities to constrain receptor mobility or spine remodeling. In this sense, PNNs are better understood as a family of local extracellular phenotypes assembled from common molecular principles but adapted to specific circuit demands (Fawcett et al., 2022).

PNNs are highly selective in their cellular localization and are especially enriched around subsets of inhibitory GABAergic interneurons, most prominently fast-spiking PV cells in cortex and hippocampus (Balmer, 2016; Ueno et al., 2017; Yamada and Jinno, 2017). This preferential association is one of the most consequential features of PNN biology, because it links extracellular specialization directly to circuit logic. PV interneurons are key regulators of perisomatic inhibition, temporal precision, and oscillatory synchronization. Their selective enwrapment by condensed ECM therefore implies that extracellular architecture is functionally tied to inhibitory gain control and critical-period regulation. Yet this association is not absolute. PNNs also surround excitatory neurons in selected regions, particularly hippocampal CA2, and can be present around other interneuron subtypes, including calbindin-positive neurons in certain structures such as the amygdala (Carstens et al., 2016; McDonald et al., 2018). Thus, PNN localization reflects physiological role rather than a simple inhibitory-excitatory dichotomy. Nets accumulate where long-term stabilization of synaptic input, firing pattern, and microenvironmental insulation are most needed (Celio et al., 1998; Carulli et al., 2006; Fawcett et al., 2022).

However, a mature account of neural ECM organization cannot, be reduced to somato-dendritic nets alone. In addition to diffuse ECM in the neuropil and PNNs around somata and proximal dendrites, matrix assemblies are present in perisynaptic spaces, at the axon initial segment (AIS), and in the specialized ECM of nodes of Ranvier (Fawcett et al., 2019). These domains are related, but not identical, and they differ in molecular composition, developmental timing, and physiological role. Perisynaptic ECM occupies the immediate extracellular environment of excitatory and inhibitory synapses and includes HA, lecticans, link proteins, and glycoproteins positioned in close relation to active zones and postsynaptic membranes (Wallace, 1989; Duncan et al., 2021). This matrix is not simply a developmental remnant. It exerts nanoscale control over receptor movement and transmitter signaling. Frischknecht and colleagues demonstrated that enzymatic perturbation of brain ECM increases extrasynaptic mobility of AMPA receptors and facilitates exchange between synaptic and extrasynaptic receptor pools, thereby showing that ECM is a direct regulator of synaptic compartmentalization and short-term plasticity (Frischknecht et al., 2009). Similarly, HA influences hippocampal synaptic plasticity through effects on postsynaptic L-type calcium channels, emphasizing that ECM molecules participate actively in the induction of plastic change (Kochlamazashvili et al., 2010).

The AIS-associated ECM deserves special emphasis because it extends the relevance of matrix biology from synaptic modulation to spike initiation itself (Brückner et al., 2006; Baidoe-Ansah et al., 2025). Classical PNNs are most conspicuous around soma and proximal dendrites, but extracellular coats containing aggrecan, tenascin-R, and hyaluronan also extend to the AIS in selected neurons, and the perinodal ECM at nodes of Ranvier represents another highly organized HA-rich specialization (Fawcett et al., 2019). These domains may appear in developmental sequence before fully mature somatodendritic nets and can be retained under conditions in which somatic PNNs are attenuated, indicating a degree of structural autonomy (Oohashi et al., 2015; Stevens et al., 2021; Baidoe-Ansah et al., 2025). This result has important mechanistic consequences. The site at which synaptic integration is converted into action potential initiation is precisely where local extracellular composition could influence membrane excitability, ionic microenvironment, channel clustering, and conduction fidelity. Studies of ankyrin-R further strengthen this view by showing that membrane-cytoskeletal anchoring is intimately linked to PNN organization and to the excitability of fast-spiking interneurons (Stevens et al., 2021). In other words, ECM specialization is not confined to the “outside” of neurons in a generic sense; it is anatomically targeted to functionally decisive membrane domains.

### 2.3 Cellular sources and molecular assembly of ECM

ECM molecules in the CNS are produced by both neurons and glial cells (see Figure 1). Neurons supply key components directly, including HAS-associated HA backbones and several proteoglycan or scaffold-associated molecules (Nguyen et al., 2020; Dzyubenko and Hermann, 2025; Hodebourg et al., 2025). The capacity of neurons to drive core net assembly is underscored by *in vitro* work showing that forced expression of hyaluronan synthase together with link protein can support formation of a PNN-like matrix and recruitment of aggrecan. At the same time, glia contributes substantially to the extracellular milieu. Astrocytes, oligodendroglial lineage cells, and other non-neuronal elements provide matrix molecules, regulate local ionic and metabolic conditions, and shape the environments in which assembly and remodeling occur (Dzyubenko and Hermann, 2025; Nakajo et al., 2026). Microglia add yet another level of control: they do not function primarily as the architects of the mature lattice, but they can locally remodel extracellular material around synapses in an activity-dependent manner (Nguyen et al., 2020). Nguyen and colleagues showed that microglial remodeling of ECM promotes synapse plasticity, reinforcing the idea that neural extracellular architecture is an emergent property of neuron–glia interaction and continuous circuit activity.

**Figure 1:**
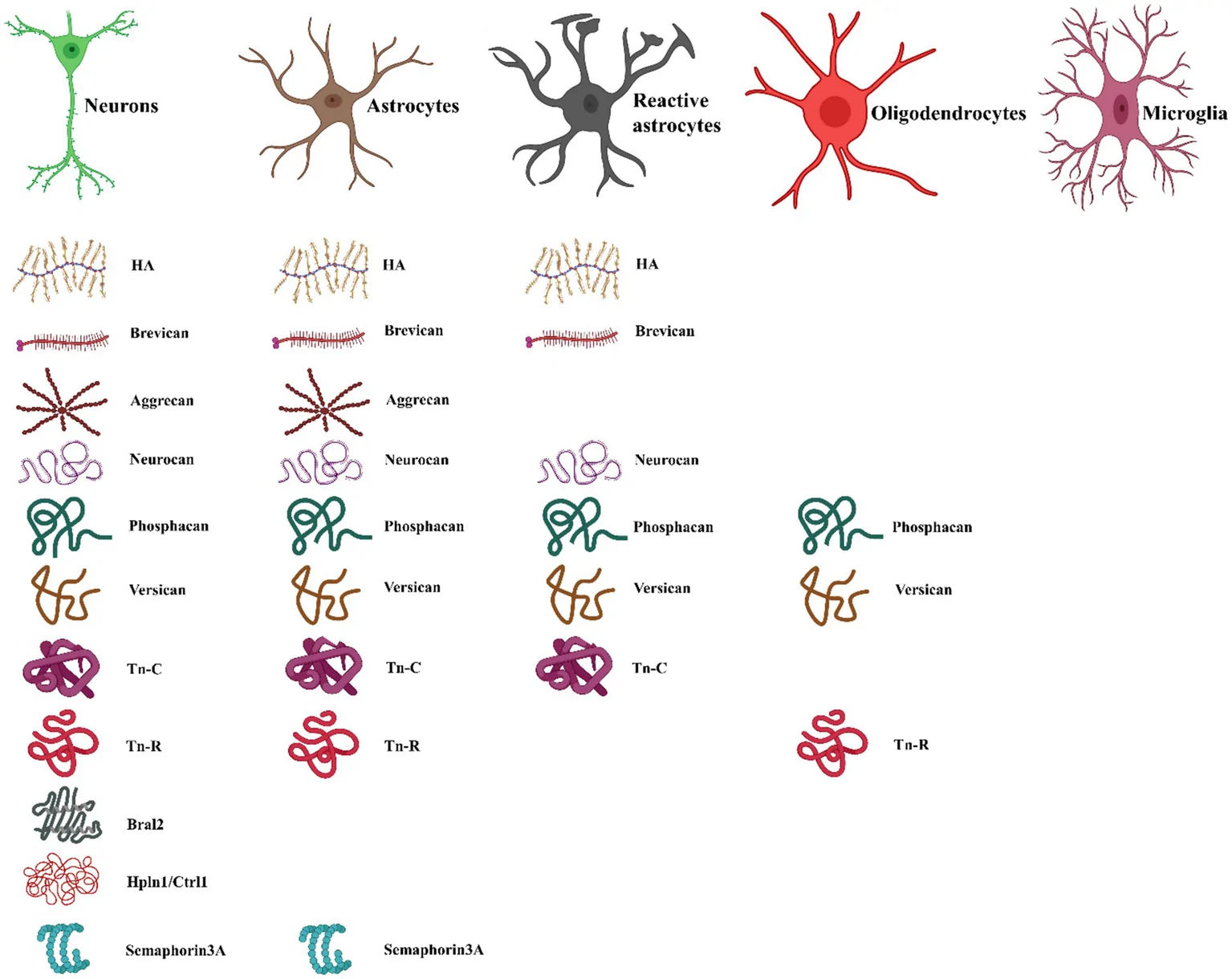
Expression of ECM components by brain cells.

This shared cellular origin helps explain a critical feature of the mature matrix: it is stable, but not fixed. The adult ECM is often described as long-lived, and in many respects this is true. PNNs are highly cross-linked and can be remarkably resistant to dissociation (Dityatev et al., 2007). Yet “stable” should not be confused with “immutable.” Matrix components are continuously subject to activity-dependent proteolysis, endocytic recycling, resurfacing, and local replacement (Dankovich et al., 2021). Metalloproteinases and related extracellular enzymes can rapidly alter ECM composition near active synapses, and this local remodeling may be sufficient to alter receptor mobility, spine dynamics, and inhibitory tone without requiring wholesale destruction of the net (see Figure 2; Sanchez et al., 2024; Nguyen et al., 2020). This semi-stable state is central to the logic of the system. A completely rigid matrix would preserve circuit identity at the expense of learning and adaptation; a permanently permissive matrix would destabilize mature computation. The adult neural ECM therefore occupies an intermediate position between persistence and plasticity (Dityatev et al., 2007; Nguyen et al., 2020; Wiera and Mozrzymas, 2021).

**Figure 2:**
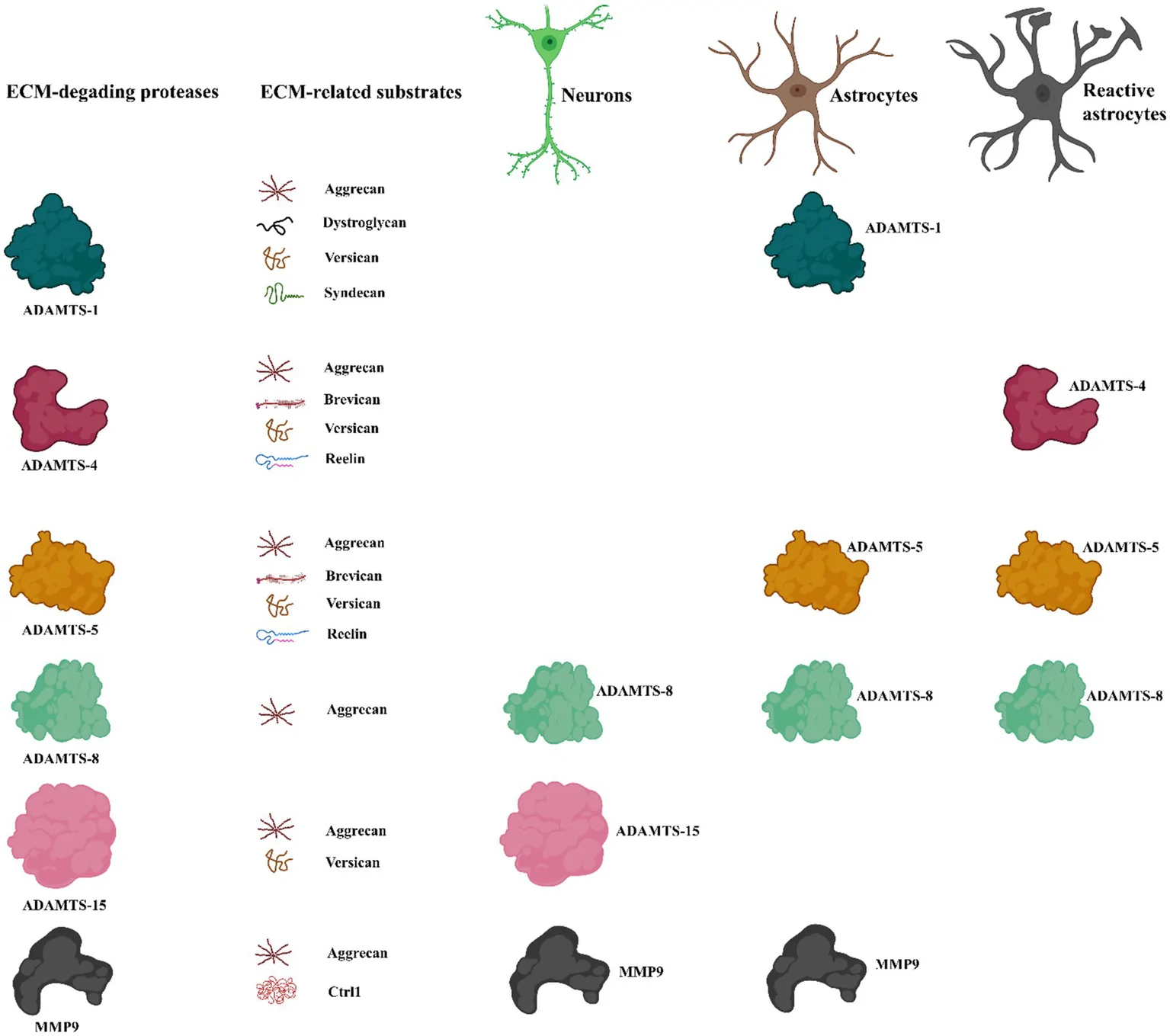
Expression of ECM-degrading proteases by brain cells.

### 2.4 Developmental maturation of ECM and PNNs

Developmentally, the ontogeny of the neural ECM reveals a transition from a comparatively permissive juvenile environment to a more structured and restrictive adult extracellular state (Yamaguchi, 2000; Oohashi et al., 2015). During embryonic and early postnatal development, the CNS ECM supports proliferation, migration, neurite extension, synaptogenesis, and experience-dependent circuit refinement. Developmental matrix is enriched in molecules and molecular states compatible with growth and reorganization (Yamaguchi, 2000; Gu et al., 2009; Berretta, 2012). Over time, however, both composition and organization change. Neurocan and versican are more prominent during earlier developmental stages, whereas aggrecan and brevican assume increasing importance in the mature brain. In parallel, ECM molecules become progressively organized into condensed pericellular lattices (Oohashi et al., 2015; Balashova et al., 2019). In rodents, mature PNNs generally emerge during the first postnatal weeks and increase in parallel with the maturation of inhibitory circuitry and the decline of critical-period plasticity (Yamaguchi, 2000; Fawcett et al., 2022). Thus, adult-like circuit stabilization is accompanied by a phase transition in extracellular organization itself, not merely by an increase in total matrix abundance.

One of the clearest demonstrations that matrix assembly matters as much as matrix abundance comes from the work on cartilage link protein Crtl1/proteoglycan link protein 1 (Hapln1) (Carulli et al., 2010). Carulli and colleagues showed that Crtl1 (now termed Hapln1 in mouse literature) is upregulated during development as PNNs form, and that animals lacking Crtl1 exhibit attenuated nets despite relatively preserved total CSPG levels and unchanged overall glycan sulfation patterns. Crucially, these animals retain juvenile-like ocular dominance plasticity, remain vulnerable to deprivation-induced changes in visual acuity, and display axonal sprouting in partially denervated sensory pathways. Thus, the biological effect of ECM cannot be inferred from the bulk presence of its molecules in tissue extracts. What matters is whether those molecules are assembled into the specific supramolecular configuration that constitutes a mature PNN.

Moreover, developmental layer of control is encoded in the sulfation profile of chondroitin sulfate chains (Maeda et al., 2011). CSPG function is determined not only by core protein identity but also by the arrangement of sulfate groups along GAG chains. This “sulfation code” changes over development and aging, thereby altering ligand binding and the permissiveness of the ECM (Dzyubenko et al., 2016). Miyata and colleagues showed that a developmental increase in the 4-sulfation/6-sulfation ratio contributes to critical-period closure in the visual cortex, and that increasing chondroitin 6-sulfation prevents condensation of CSPGs into mature PNNs around PV interneurons. Importantly, this altered sulfation state also interferes with Otx2 accumulation and with electrophysiological maturation of PV cells (Miyata et al., 2012; Fawcett et al., 2022). Thus, ECM ontogeny is both structural and chemical. The maturing brain does not merely build more matrix; it builds a biochemically more restrictive matrix, one increasingly suited to circuit stabilization and less permissive to unconstrained rewiring.

PNN ontogeny is inseparable from neuronal maturation. Nets do not simply appear after neurons have completed development; they participate in defining the mature state of the neurons they surround (Beurdeley et al., 2012). This is especially evident for PV interneurons, in which PNN accumulation correlates with maturation of fast-spiking properties, stabilization of synaptic contacts, and the closure of heightened developmental plasticity. The emerging net reinforces the mature phenotype by concentrating ligands such as OTX2, by restricting receptor mobility, by organizing ionic and molecular microenvironments, and by anchoring the interactions between extracellular scaffolds and membrane-cytoskeletal elements such as ankyrin-R (Stevens et al., 2021; Alzarea, 2026). The result is a reciprocal system in which neuronal activity shapes extracellular assembly, and extracellular assembly in turn constrains the future range of neuronal responses. Such reciprocity is one of the defining principles of neural ECM biology.

In addition, the mature PNN should also be understood as a molecular address system that recruits andretains regulatory ligands. OTX2 provides the clearest example (Beurdeley et al., 2012). This homeoprotein binds selectively to defined PNN-associated chondroitin sulfate motifs, is internalized by PV interneurons, and contributes both to their maturation and to maintenance of the adult PNN state. Neuronal pentraxin 2 (Nptx2/Narp) provides another important layer of regulation (Chang et al., 2010). It accumulates at excitatory synapses on PV interneurons, promotes AMPA receptor clustering, and interacts functionally with the matrix; more recent work shows that Nptx2 binds both HA and chondroitin sulfate motifs and can enhance PNN formation (Van’t Spijker et al., 2019). Sema3A adds a further dimension by linking the extracellular scaffold to guidance signaling and potentially to matrix rigidity or synaptic accessibility (Fawcett et al., 2022). Together, these findings argue strongly against the view that PNNs are inert wraps around neurons. They are biochemically active extracellular platforms that selectively capture, concentrate, and spatially present instructive molecules to particular neuronal classes.

## 3 ECM/PNNs in synapses and neural plasticity

### 3.1 Receptor mobility and synaptic transmission in ECM/PNNs

Synaptic transmission does not occur in a chemically neutral void, but within a highly organized extracellular compartment (Dityatev and Schachner, 2003; Dityatev and Rusakov, 2011). In the mature central nervous system, synapses are embedded in a molecular meshwork composed principally of HA, CSPGs, HSPGs, link proteins, tenascins, thrombospondins, and a broader set of diffusible or matrix-bound glycoproteins. Around selected neuronal populations, particularly fast-spiking PV interneurons, this matrix condenses into PNNs, which surround the soma and proximal dendrites and extend into the immediate perisynaptic environment (Lavekar et al., 2023). Moreover, the extracellular milieu is part of the synaptic apparatus itself. It regulates diffusion of ions and neurotransmitters, constrains receptor access to ligands, stabilizes synaptic geometry, and shapes how enduring structural change can be expressed in mature circuits (Melrose et al., 2021). In this sense, the ECM is not a peripheral scaffold appended to the synapse after the fact; it is an active determinant of synaptic operating conditions and a core component of what has been described as the “tetrapartite synapse” or, in broader usage, the extracellularly framed synaptic unit (Dityatev and Rusakov, 2011).

This framework is strengthened when one considers the anatomical heterogeneity of neural ECM. Classical neuropathological descriptions emphasized the conspicuous lattice-like morphology of PNNs, but later work made clear that these nets are only the most visible expression of a wider extracellular continuum (Dityatev and Schachner, 2003; Dityatev et al., 2014). Beneath and beyond the perisomatic net lies a diffuse interstitial matrix, a perisynaptic matrix closely associated with excitatory and inhibitory contacts, and additional specializations at the axon initial segment and nodes of Ranvier (Fawcett et al., 2019). The distinction matters because experimental manipulation of “PNNs” often perturbs more than one extracellular compartment. Enzymatic digestion of chondroitin sulfate or HA, or genetic perturbation of link proteins and matrix organizers, may alter perisomatic lattice integrity, perisynaptic receptor microenvironments, extracellular space geometry, and the long-term physiological identity of inhibitory neurons at the same time (Fawcett, 2015; Fawcett et al., 2022). Therefore, different matrix compartments regulate distinct aspects of synaptic function, and similar manipulations may yield superficially similar increases in plasticity through mechanistically different routes.

One of the clearest demonstrations that extracellular structure directly enters the molecular machinery of synaptic physiology came from the work of Frischknecht and colleagues, who showed that enzymatic attenuation of brain ECM increases the lateral mobility of AMPA receptors in the neuronal membrane and enhances exchange between synaptic and extra-synaptic receptor pools (Frischknecht et al., 2009). In addition, ECM removal also altered short-term synaptic behavior, including an increase in paired-pulse ratio, indicating that the surface compartments formed by the extracellular matrix have measurable consequences for synaptic function. The importance of this finding lies in what it dislodged: receptor trafficking could no longer be treated solely as an intracellular problem of endocytosis, exocytosis, and postsynaptic scaffold anchoring. Synaptic receptors move in a membrane landscape whose effective topology is partly imposed from the outside. By forming extracellular diffusion barriers and compartment boundaries, the mature ECM helps preserve synaptic identity and dampens excessive receptor overmobility. This stabilizing role is especially relevant once developmental circuit refinement has largely ended, because adult information processing depends not only on the capacity for rapid receptor exchange, but also on the maintenance of persistent synaptic signatures across repeated rounds of activity (Kwok and Dityatev, 2026).

### 3.2 Structural plasticity and dendritic spine dynamics in ECM/PNNs

Extracellular regulation of plasticity is not generic, but highly molecule-specific. Early work by Bukalo and colleagues demonstrated that enzymatic removal of chondroitin sulfate and constitutive deficiency of tenascin-R do not produce interchangeable effects in the hippocampus; rather, they differentially affect several forms of synaptic plasticity (Bukalo et al., 2001). This finding argues against reducing the ECM to a single inhibitory principle. The same lesson emerges from tenascin-C deficiency, which impairs L-type voltage-dependent calcium channel (L-VDCC)-dependent forms of hippocampal synaptic plasticity without grossly deranging CNS structure (Evers et al., 2002; Dityatev and Schachner, 2003). Such results indicate that extracellular molecules engage plasticity through distinct biochemical routes rather than through a uniform effect of bulk tissue “stiffness” or steric hindrance. The mature ECM is therefore better understood as a molecularly heterogeneous signaling platform whose various constituents differentially regulate receptor clustering, calcium entry, adhesion signaling, cytoskeletal coupling, and the probability that particular forms of long-term potentiation (LTP) or long-term depression (LTD) can be induced and maintained (Dzyubenko and Hermann, 2023).

This molecular specificity is illustrated particularly elegantly by studies of HA, the principal glycosaminoglycan scaffold of the neural ECM. Although HA was long regarded mainly as a space-filling polymer, Kochlamazashvili and colleagues demonstrated that it also exerts a highly specific control over hippocampal synaptic plasticity by modulating postsynaptic L-type Ca^2+^ channels. Enzymatic removal of HA reduced nifedipine-sensitive calcium currents, decreased dendritic and spine Ca^2+^ transients mediated by L-VDCCs, and abolished an L-VDCC-dependent component of CA3-CA1 LTP; notably, these effects were reversed by exogenous HA (Kochlamazashvili et al., 2010; Melrose et al., 2021). This study was decisive because it linked extracellular matrix biology directly to one of the canonical intracellular gateways of long-term synaptic change. By tuning calcium entry, HA reaches into the core transduction cascade through which synapses convert patterned depolarization into persistent biochemical modification (Fawcett et al., 2022).

The relevance of ECM specialization becomes even more pronounced in inhibitory microcircuits, where PNNs are concentrated around PV interneurons (Miyata and Kitagawa, 2017). These interneurons are essential for feedforward and feedback inhibition, temporal precision, oscillatory synchronization, and the stabilization of excitation–inhibition balance. For instance, Cardin and colleagues provided causal *in vivo* evidence that activation of fast-spiking interneurons selectively amplifies gamma-frequency activity and that the phase of sensory input relative to the induced gamma cycle determines the amplitude and temporal precision of cortical responses (Cardin et al., 2009). In parallel, Sohal and colleagues showed that suppressing PV interneurons reduces gamma oscillations, whereas driving them is sufficient to generate gamma rhythmicity and can improve cortical information transmission by reducing noise and amplifying signals (Sohal et al., 2009). Balmer (2016) found that ChABC-mediated attenuation of CSPGs-rich nets reduced the gain of spike output, increased spike threshold/delay, and reduced excitability in fast-spiking populations, including PV-expressing cortical neurons. It is therefore unlikely that the dense matrix investing PV cells is an incidental histological ornament of maturation. A more plausible view is that PNNs are part of the machinery that consolidates and maintains the mature PV-cell phenotype. Beurdeley and colleagues showed that PNNs on PV cells selectively capture OTX2 and that this interaction is necessary for the persistent regulation of critical period plasticity in visual cortex (Beurdeley et al., 2012; Fawcett, 2015). Moreover, the glycosaminoglycan component of nets, especially specific sulfation motifs such as CS-E, is central to this process (Fawcett et al., 2022). Thus, PNNs do not merely inhibit new synaptic growth in a nonspecific fashion; they also maintain a mature inhibitory state through selective molecular sequestration. This distinction matters. The closure of a critical period is not simply the imposition of extracellular rigidity, but the establishment of a stabilized physiological identity in key inhibitory neurons.

Genetic evidence reinforces the idea that organized net assembly, not merely the presence of individual CSPGs, is a decisive event in the regulation of adult plasticity. Carulli and colleagues showed that mice lacking the link protein Crtl1/Hapln1 display attenuated PNNs while retaining overall CSPG levels and glycan sulfation patterns, yet these animals preserve juvenile-like ocular dominance plasticity and remain sensitive to visual deprivation (Carulli et al., 2010). This finding is important for two reasons. First, it establishes that the architecture of matrix assembly itself has functional significance. Second, it suggests that the transition from juvenile plasticity to adult stability depends on the supramolecular organization of ECM components into nets rather than simply on their absolute abundance. Related work on sulfation patterns reaches a similar conclusion from another angle: Miyata and colleagues showed that increased chondroitin 6-sulfation maintains persistent cortical plasticity, indicating that qualitative changes in glycan structure, not just matrix quantity, can shift the threshold for adult circuit modification (Miyata et al., 2012). Mature ECM is therefore best viewed as a biochemical code embedded in a supramolecular structure. Both the code and the structure matter for determining whether a circuit remains open to reconfiguration or becomes stabilized against further change (Fawcett, 2015).

The synaptic consequences of PNN manipulation are region- and circuit-specific rather than uniformly facilitatory. This point deserves emphasis because “PNN removal reopens plasticity” is accurate only at a very general level. In the hippocampus, most studies summarized by Fawcett and colleagues indicate that degradation or attenuation of PNNs in CA1 reduces LTP (Fawcett et al., 2022), whereas other work has shown that PNNs surrounding CA2 pyramidal neurons suppress potentiation of excitatory synapses in that region and that disruption of those nets allows LTP to emerge (Carstens et al., 2016). Likewise, PNNs appear to restrict LTD induction in adult CA1 (Khoo et al., 2019). The lesson is not inconsistency, but specificity. Different circuits use extracellular constraints for different computational ends. In some regions, nets preserve the gain and timing of already optimized synapses; in others, they maintain a deliberate resistance to modification that itself is functionally important (Wingert and Sorg, 2021). The electrophysiological impact of PNN attenuation therefore may depend on neuronal class, synapse type, developmental state, and the exact matrix compartment affected.

An additional layer of regulation concerns homeostatic plasticity. Hebbian plasticity alone cannot explain how neuronal networks preserve stable baseline function while undergoing repeated activity-dependent modification. Here, extracellularly localized molecules linked to PV-cell synapses become especially relevant. Chang and colleagues showed that Narp, an immediate-early gene product encoding a secreted neuronal pentraxin, accumulates preferentially at excitatory synapses onto PV interneurons and is required for activity-dependent homeostatic strengthening of those inputs (Chang et al., 2010). Although Narp is not itself a canonical structural component of the classical PNN backbone, it exemplifies a broader principle: extracellularly organized synaptic proteins can recruit AMPA receptors and help recalibrate inhibitory output when network activity rises. This mechanism has obvious systems-level importance (Dityatev and Rusakov, 2011). By strengthening excitatory drive onto PV interneurons, networks can enhance inhibitory restraint and re-establish excitation–inhibition balance. It is therefore increasingly difficult to separate ECM/PNN biology from homeostatic stabilization. The extracellular compartment is not only where synaptic weights are expressed; it is also where some of the compensatory mechanisms that protect network equilibrium are organized (Frischknecht et al., 2014).

### 3.3 Activity-dependent extracellular proteolysis in ECM/PNNs

The dynamic character of adult neural ECM also depends on tightly regulated proteolysis (Tsilibary et al., 2014). The mature brain matrix is not inert; it is continuously surveilled and locally remodeled by extracellular proteases, especially matrix metalloproteinases (MMPs) and related enzymes (De Luca and Papa, 2017). Activity-dependent proteolysis can loosen extracellular constraints, alter receptor accessibility, reshape adhesion complexes, expose cryptic binding sites, and generate bioactive fragments. The importance of this process lies in its locality. Global dissolution of mature ECM would endanger circuit identity, but spatially restricted proteolysis provides a mechanism by which plasticity can be permitted at selected synapses without destabilizing the entire network (Wiera and Mozrzymas, 2015). Wiera and Mozrzymas (2021) highlighted that this principle applies not only to excitatory synapses, where the evidence is strongest, but also to inhibitory synapses, an area of growing significance given the centrality of inhibitory timing and oscillatory coordination to memory processing and disease vulnerability. Protease-mediated ECM remodeling thus appears to solve a major biological problem: how to reconcile the need for extracellular stability with the equally real need for adult synapses to remain modifiable under appropriate physiological demand.

In addition, the work of Nguyen and colleagues demonstrated that neuron-derived IL-33 instructs microglial engulfment of extracellular matrix components in the adult hippocampus, thereby promoting dendritic spine plasticity, synaptic remodeling, and the precise consolidation of long-term memories (Nguyen et al., 2020). The conceptual advance of this work is substantial. Microglia are not merely phagocytes that clear debris or eliminate weakened synapses; they are active editors of the perisynaptic matrix. By locally removing or restructuring ECM components near active synapses, microglia may create transient windows in which structural remodeling can occur without requiring indiscriminate degradation of mature extracellular architecture (Cangalaya et al., 2024; Wareham and Calkins, 2025). This interpretation fits well with the broader shift in the field from a neuron-centric model of plasticity to one in which glia, extracellular matrix, and synapses form an integrated functional ensemble. It also helps explain why adult plasticity can be highly focal and experience-dependent despite the presence of apparently restrictive matrix structures in the mature brain.

From this perspective, the neural ECM and PNNs are best understood not as blanket suppressors of plasticity, but as regulators of plasticity permissiveness, localization, and persistence (Melrose et al., 2021; Fawcett, 2015; Fawcett et al., 2022). They restrict receptor over-mobility, preserve extracellular compartmentation, support the mature inhibitory phenotype of PV interneurons, and raise the threshold for indiscriminate structural reorganization. At the same time, because they remain susceptible to local proteolysis, glial editing, and alterations in glycan sulfation, they permit defined forms of plasticity to occur under selected physiological or pathological conditions. Such a model resolves an older false dichotomy. The ECM is neither simply permissive nor simply inhibitory. It can facilitate some forms of long-term change while constraining others, depending on the molecular component involved, the brain region affected, and the computational role of the circuit under study.

### 3.4 Developmental compensation versus adult manipulation in ECM and PNNs

A major methodological limitation in interpreting ECM genetics is the difference between constitutive and adult-restricted manipulation. ECM and PNN molecules participate in development, precursor behavior, synapse maturation and critical-period regulation; deletion from embryonic or early postnatal stages can therefore alter the trajectory by which the adult circuit is built. For instance, tenascin-C deletion changes oligodendrocyte precursor migration and proliferation and is accompanied by later changes in programmed cell death that could partly normalize cell numbers (Garcion et al., 2001). Thus, an apparently mild adult phenotype does not necessarily indicate that the molecule is dispensable.

Notably, this caveat has now been demonstrated directly for PNN genes. Germline PV-cell Acan deletion eliminated WFA-defined nets but produced limited changes in adult PV-cell electrophysiology and ocular-dominance plasticity, whereas adult AAV-mediated deletion increased plasticity; increased *Ncan* and *Tnr* expression in the germline model supported compensation (Grødem et al., 2025). Baidoe-Ansah et al. (2026) similarly found cognitive and synaptic consequences after adult regional Ncan depletion that were absent in constitutive *Ncan* knockout mice.

It is reasonable to consider that constitutive knockout, adult conditional/regional knockout and enzymatic digestion are complementary but non-equivalent approaches. Constitutive genetics offers molecular specificity but is vulnerable to developmental compensation; adult viral/conditional approaches improve temporal and regional specificity; and enzymes such as ChABC provide rapid manipulation but alter CS-rich ECM broadly. Strong mechanistic conclusions are most persuasive when convergent results are obtained across more than one of these approaches.

## 4 ECM/PNNs during the memory stage and circuit- and task-dependent effects

In the mature brain, durable memory depends on the coordinated interaction of intracellular signaling cascades, synaptic architecture, inhibitory circuit timing, and the extracellular environment that stabilizes these relationships across time (Marco, 2022). Within this framework, the neural ECM and PNNs are not incidental structures that become relevant only after learning has already occurred. Rather, they help define the conditions under which experience-dependent synaptic modifications can be encoded, consolidated, maintained, updated, or extinguished (Melrose et al., 2021; Fawcett et al., 2022). This point is especially important in adult circuits, where the core biological problem is no longer simply how to generate plasticity, but how to preserve selected circuit changes without permitting uncontrolled rewriting of established network structure. ECM and PNN function should therefore be interpreted not as the opposite of plasticity, but as one of the mechanisms by which plasticity becomes selective, durable, and circuit-appropriate (Dityatev and Rusakov, 2011; Fawcett et al., 2022).

At the mechanistic level, the relevance of the ECM to memory begins well before overt behavior is measured. Brain ECM regulates synaptic plasticity by controlling receptor mobility at the neuronal surface, shaping extracellular diffusion, restricting the access of signaling molecules to synaptic microdomains, and modulating ion channel function and neuronal excitability (Senkov et al., 2014; Cingolani et al., 2019). The importance of these extracellular constraints is illustrated by the finding that hyaluronan is not merely a structural component of mature PNNs but also directly influences hippocampal synaptic plasticity by modulating postsynaptic L-VDCCs (Evers et al., 2002; Dityatev and Schachner, 2003). Because calcium entry is central to the induction threshold and direction of long-term synaptic modification, such observations place ECM regulation closes to the molecular core of learning-related plasticity rather than at its periphery (Kochlamazashvili et al., 2010; Fawcett et al., 2022). In the same general sense, ECM organization influences AMPA receptor mobility, synaptic responsiveness, and local protease-dependent remodeling, indicating that extracellular architecture contributes to the conversion of transient activity into lasting synaptic bias (Boudkkazi et al., 2023; Nakajo et al., 2026). Memory formation depends precisely on this conversion.

A central implication follows. ECM and PNNs do not determine memory content in any simple representational sense. Instead, they determine whether a given circuit remains permissive to synaptic updating, how restricted such updating is in space and time, and how resistant the resulting network state becomes to subsequent destabilization (Frischknecht et al., 2009; Melrose et al., 2021; Fawcett et al., 2022). In mature networks, PNNs are better viewed as extracellular gatekeepers of post-acquisition malleability. They do not abolish plasticity; they regulate when, where, and to what extent plasticity remains accessible after learning.

In addition, PNNs do not uniformly promote memory persistence or uniformly suppress memory. Rather, they regulate the range and stability of plastic changes in a circuit- and task-dependent manner. One of the most decisive demonstrations of this principle came from studies of fear extinction (Gogolla et al., 2009). In the amygdala, enzymatic degradation of PNN-associated CSPGs makes subsequently acquired fear memories susceptible to erasure by extinction, supporting a stabilizing role in that circuit and memory phase (Gogolla et al., 2009). In contrast, genetic or local enzymatic attenuation of nets can enhance long-term object-recognition memory and facilitate LTD in perirhinal cortex (Romberg et al., 2013). Rowlands et al. (2018) likewise reported improved object recognition after brain-wide aggrecan targeting. Conversely, PNN disruption in hippocampus or medial prefrontal cortex impairs specific fear-memory components (Hylin et al., 2013), and Liu et al. (2023) found that CA1 PNN elimination reduces PV-neuron firing and GABA release and impairs long-term contextual fear-memory retention. These apparently divergent outcomes are biologically informative rather than contradictory. The consequence of ECM/PNN attenuation depends on which circuit is recruited, whether the task requires flexible updating versus stable retention, the stage of memory being tested (acquisition, consolidation, retrieval, extinction or reconsolidation), and the timing and spatial extent of the manipulation. Thus, increased plasticity can improve performance when flexibility is the limiting computation but impair performance when stable inhibitory timing or trace preservation is required.

This logic extends naturally to appetitive and addiction-related learning (Knapska et al., 2013; Smith et al., 2015; Ray et al., 2022). Drug-associated memories are often not remarkable because they are acquired more efficiently than other forms of memory, but because they persist with abnormal tenacity and remain highly susceptible to reactivation by cues, context, or stress (Sorg, 2012). Work on the amygdala has shown that depletion of PNNs can enhance the erasure of drug memories under extinction-like conditions (Xue et al., 2014). The importance of this finding lies in the distinction between memory expression and memory lability. PNN attenuation does not necessarily abolish the capacity to express a learned association immediately; rather, it can re-open the trace to subsequent modification and weaken its resistance to behavioral updating. In pathological states such as addiction, this becomes highly relevant. The same extracellular specializations that help preserve appropriate circuit identity under physiological conditions may also stabilize maladaptive memory states once they are consolidated (Xue et al., 2014; Ray et al., 2022). The therapeutic appeal of ECM-targeting strategies follows directly from this tension between protection and flexibility.

Notably, PNN/ECM attenuation has phase-, circuit-, and timing-dependent effects on memory rather than a uniformly beneficial or detrimental effect. Enzymatic matrix digestion can enhance reversal learning and can increase the susceptibility of fear or drug memories to extinction-based updating (Gogolla et al., 2009; Happel et al., 2014; Xue et al., 2014; Duncan et al., 2019). In contrast, PNN disruption can impair fear learning and consolidation in hippocampal and prefrontal circuits, but the behavioral consequence is strongly dependent on the task, timing, and brain region targeted; in other paradigms, such as object-based memory or diet-induced memory dysfunction (Reichelt et al., 2026), PNN remodeling may instead facilitate specific forms of plasticity (Banerjee et al., 2017; Thompson et al., 2018; Liu et al., 2023). These opposing effects that are dependent on task and timing are further illustrated by Reichelt et al. (2026), who showed within the same hippocampal model that PNN degradation after an established diet-induced memory deficit restored performance, whereas degradation before the insult exacerbated impairment. Mechanistically, these bidirectional behavioral effects are consistent with the contribution of PNNs to PV-interneuron physiology and stable network dynamics: PNN removal can alter fast-spiking neuronal excitability, inhibitory network activity, and the stability of neuronal representations (Balmer, 2016; Lensjø et al., 2017; Shi et al., 2019; Christensen et al., 2021). Accordingly, the behavioral consequence of ECM/PNN remodeling depends on when and where the matrix is modified and on the specific memory operation being tested.

Aging further clarifies this point (Zhu et al., 2025). Cognitive decline in aging is unlikely to reflect a simple absence of plasticity-related machinery. At least in part, it may reflect the progressive accumulation of extracellular states that favor preservation of old circuit configurations at the expense of adaptive updating. In that setting, the problem is not that the aged brain lacks ECM, but that it may possess too much of the wrong kind of extracellular stabilization, whether in overall PNN density, in sulfation pattern, or in the balance between permissive and inhibitory glycosaminoglycan species. For instance, Chondroitin-sulfate function cannot be inferred from total CSPG abundance alone because sulfation pattern changes ligand binding and the permissive properties of the matrix. Miyata and colleagues showed that the developmental increase in the CS 4S/6S ratio is required for normal PNN condensation, Otx2 accumulation, PV-interneuron maturation and closure of ocular-dominance plasticity (Miyata et al., 2012). Aging introduces a related but distinct shift: Yang and colleagues found an age-related reduction in permissive C6S, early memory deficits after Chst3 deletion, and rescue of aged memory/plasticity after restoring C6S or attenuating CS-rich ECM (Yang et al., 2021). This is why ECM-targeting interventions can improve cognition under some aging and disease conditions: they may restore an extracellular range within which established circuit identity is retained but new experience can still meaningfully reshape synaptic relationships (Hendrickson et al., 2024; Zhu et al., 2025). Thus, the aging brain confronts a particularly delicate extracellular trade-off: enough matrix organization to preserve fidelity, but not so much that learning, updating, and compensation become biologically inaccessible.

The memory study also shows that extracellular regulation is not reducible to PNNs alone. Reelin is particularly important in this respect because it links extracellular signaling to dendritic spine maintenance, synaptic efficacy, and hippocampus-dependent learning (Rogers and Weeber, 2008). Specifically, Reelin is a secreted extracellular glycoprotein that remains functionally active in the adult brain and modulates excitatory synapses through ApoER2/VLDLR-Dab1 signaling. Beffert and colleagues showed that ApoER2 is present in excitatory postsynaptic densities, forms a functional complex with NMDA receptors, and is required for Reelin-dependent enhancement of NMDA-receptor phosphorylation and hippocampal LTP (Beffert et al., 2005). Durakoglugil and colleagues further demonstrated that Reelin can oppose Aβ-induced suppression of NMDA-receptor function and LTP through Src-family kinase signaling (Durakoglugil et al., 2009). Conceptually, Reelin prevents an overly narrow view in which the ECM is understood only as a stabilizing shell around mature inhibitory neuron. Some extracellular molecules actively facilitate memory-related plasticity rather than constrain it. Reelin enhances synaptic efficacy, supports structural maturation of dendritic spines, and remains functionally relevant in the adult brain (Stranahan et al., 2013; Santana and Marzolo, 2017). In disease-relevant contexts, supplementation of Reelin can rescue synaptic and behavioral deficits, indicating that extracellular insufficiency may become rate-limiting for cognitive function under pathological conditions (Rogers et al., 2013). Or, memory depends on a distributed extracellular system composed of both stabilizing and permissive components. PNN-associated CSPGs often bias mature networks toward persistence, whereas signaling molecules such as Reelin can enhance the plastic potential required for encoding and adaptive compensation (Pujadas et al., 2014; Fawcett et al., 2022).

Taken together, this context dependence becomes especially relevant in disease, where therapeutic remodeling must be timed and targeted rather than simply maximized.

## 5 ECM/PNNs in neurological disease

### 5.1 ECM/PNNs in neurodevelopmental/psychiatric disorders

The involvement of the ECM and PNNs in neurological disease follows directly from their normal physiological roles (Melrose et al., 2021; Fawcett et al., 2019). If extracellular structures stabilize synapses, preserve the mature state of inhibitory interneurons, regulate receptor mobility, shape extracellular diffusion, and delimit the circumstances under which adult circuits remain open to revision, then pathology need not arise solely from neuronal death, transmitter imbalance, or cell-autonomous molecular defects. It can also arise from pathological remodeling of the extracellular rules under which neural circuits operate (Heck et al., 2004; Tansley et al., 2022). Disease may emerge because the ECM becomes too permissive, allowing aberrant sprouting, unstable connectivity, or maladaptive rewiring (De Luca and Papa, 2016); but it may also emerge because the ECM becomes too rigid, preventing necessary adaptation, recovery, or compensation (Downs et al., 2023). A satisfactory disease model must therefore treat ECM pathology not as a passive consequence of neural injury, but as a change in the molecular and structural conditions that govern communication among neurons and glia.

This potential principle is evident in stress-related cognitive dysfunction. The effects of stress on PNNs are neither uniform nor static; they depend on developmental epoch, interval after stress, sex, brain region, and the behavioral domain being measured (Laham and Gould, 2022). Nevertheless, an important pattern emerges. Early after stress, PNNs can be reduced in a manner consistent with destabilization of inhibitory circuitry, whereas after longer intervals compensatory or excessive increases may appear, suggesting that chronic stress can drive circuits toward a maladaptively stabilized state (Spijker et al., 2020). Riga and colleagues provided particularly strong causal evidence for this interpretation (Riga et al., 2017). In a chronic depressive-like state, rats exhibited hippocampal ECM alterations together with impaired memory and deficient maintenance of CA1 LTP. Enzymatic attenuation of the hippocampal ECM restored electrophysiological and cognitive abnormalities. This study demonstrates that pathological extracellular remodeling can become part of the mechanism by which cognitive dysfunction is maintained. In other words, persistent disease states may be stabilized extracellularly, not merely intracellularly or synaptically.

The disease relevance of ECM biology is equally clear in neurodevelopmental and neuropsychiatric disorders. In Rett syndrome, fragile X syndrome, and schizophrenia-related conditions, the central problem is not simply reduced plasticity in the abstract. Rather, it is disturbed maturation and regulation of interneuron-centered circuitry, especially networks involving PV neurons whose fast firing and high metabolic load make them especially dependent on stable extracellular support (Siller and Broadie, 2012; Carstens et al., 2021; Pantazopoulos and Berretta, 2016). Fawcett and colleagues summarize evidence that Rett syndrome is associated with denser PNN organization and impaired CA2 plasticity, whereas fragile X-related phenotypes are associated with reduced PNNs and abnormal PV interneuron development (Fawcett et al., 2022). In the case of schizophrenia, postmortem and experimental studies have linked schizophrenia-spectrum disorders to reductions in PNN number and density in several regions, including the amygdala, entorhinal cortex, thalamic reticular nucleus, and prefrontal cortex (Bitanihirwe and Woo, 2014). Because these same disorders are strongly associated with PV interneuron dysfunction, impaired oscillatory coordination, and cognitive fragmentation, ECM pathology is unlikely to be incidental (Cabungcal et al., 2013). A defensible interpretation is that loss or abnormal maturation of PNNs contributes to the instability of inhibitory timing and cortical information processing that underlies cognitive and affective symptoms. Here again, the disease logic is not that reduced ECM automatically increases useful plasticity. Rather, it may render mature circuits insufficiently protected, excessively labile, and more vulnerable to oxidative or metabolic stress. That possibility is especially compelling for PV interneurons, whose physiological specialization comes at the cost of increased vulnerability when extracellular support is compromised (Fawcett et al., 2022). These examples are instructive because they show that both excessive and insufficient extracellular stabilization can be pathological, depending on the circuit and developmental stage involved.

Reelin reinforces the same point from a somewhat different angle. It is often discussed as a developmental guidance cue, but such framing is insufficient for disease biology. Reelin remains a functionally important extracellular regulator in the mature brain, affecting synaptic plasticity, inhibitory tone, and cognition (Stranahan et al., 2013). Experimental supplementation of Reelin in heterozygous reeler mice rescues aspects of sensorimotor gating, synaptic plasticity, and associative learning, indicating that at least part of the phenotype remains reversible at the level of adult extracellular signaling (Rogers et al., 2013). This suggests that ECM-related disease mechanisms are not merely static developmental residues; they remain biologically active and, in principle, therapeutically modifiable in adulthood. At the same time, such findings do not justify simplistic interventions aimed at globally increasing or decreasing ECM components. The extracellular system is regionally organized, chemically heterogeneous, and tightly coupled to cell type and disease stage (Lepelletier et al., 2017). Therapeutic relevance depends on specificity, not on indiscriminate softening of mature brain matrix.

### 5.2 ECM/PNNs in neurodegeneration and aging

Neurodegenerative disease adds a further layer of complexity. In Alzheimer’s disease, the ECM is relevant not simply because synapses degenerate, but because the extracellular environment that normally regulates and protects synaptic function is itself altered (Sethi and Zaia, 2017). Heparan sulfate proteoglycans and CSPGs participate in amyloid and tau pathology at several levels, including amyloid precursor protein processing, Aβ clearance, tau aggregation, and the extracellular spread of pathological species (Goedert et al., 1996; Scholefield et al., 2003; Holmes et al., 2013; Liu et al., 2016). Equally important, PNNs may exert a protective effect by restricting uptake or distribution of aggregated tau, yet PNNs themselves are altered in disease and may be lost through disease-associated microglial remodeling (Suttkus et al., 2016; Crapser et al., 2020). Thus, the ECM in neurodegeneration is not a bystander. It is part of the interface through which toxic protein pathology is translated into circuit dysfunction. Notably, Reelin is again central in this context. Pujadas and colleagues showed that Reelin interacts with soluble Aβ species, delays fibril formation, protects against Aβ-induced neuronal death and dendritic spine loss, and rescues recognition memory deficits in an APP mutant mouse model (Pujadas et al., 2014). These results suggests that Reelin does not merely correlate with disease severity; it can influence the kinetics of pathological aggregation and the degree to which proteotoxic stress is converted into synaptic and cognitive failure. This broadens the concept of ECM dysfunction in neurodegeneration. It is not limited to excessive stabilization or failed plasticity. It also includes loss of extracellular mechanisms that normally buffer the circuit against proteopathic injury.

At the same time, translational caution is necessary. Recent cross-species analyses have argued that AD-associated changes in PNN-linked chondroitin sulfate glycosaminoglycans in human brain are not always faithfully recapitulated in rodent models (Hendrickson et al., 2024). This matters because it suggests that disease-relevant ECM changes may be chemically and regionally specific. The relevant abnormality may not be the gross presence or absence of PNNs, but the precise sulfation pattern, maturation state, or regional glycan signature of the matrix. Such specificity may explain why proof-of-principle manipulations such as chondroitinase ABC digestion can be experimentally powerful while still offering only limited translational guidance in their crude form (Yang et al., 2024). In neurodegenerative disease, as in other disorders, the therapeutic target is unlikely to be “the ECM” in general. It is more likely to be a defined molecular feature of ECM organization that has become pathologically biased.

Furthermore, aging forms the conceptual bridge between neurodegeneration and the broader problem of declining plasticity. Over time, the brain appears to accumulate extracellular states that increasingly favor stabilization over revision. In moderation, this helps preserve mature circuit identity. In excess, it may harden networks against new learning, limit adaptive reorganization, and reduce resilience to injury or accumulating pathology. The disease logic is therefore continuous across conditions. Whether the initiating insult is chronic stress, neurodevelopmental dysregulation, amyloid pathology, or metabolic strain, dysfunction emerges when extracellular systems no longer maintain the proper balance between circuit protection and circuit adaptability (Melrose et al., 2021; Fawcett et al., 2022; Hendrickson et al., 2024). This is why ECM biology repeatedly reappears across otherwise disparate neurological and psychiatric disorders: extracellular remodeling is one of the fundamental ways by which the circuitry is able to respond appropriately to environmental changes.

These considerations also clarify why ECM and PNN pathology should not be described vaguely as evidence of “reduced plasticity”. The pathological problem differs across diseases (Siller and Broadie, 2012; Rogers et al., 2013; Pujadas et al., 2014; Pantazopoulos and Berretta, 2016; Riga et al., 2017; Carstens et al., 2021; Fawcett et al., 2022; Hendrickson et al., 2024). In depression-like states, excessive hippocampal ECM may stabilize cognitive impairment (Riga et al., 2017; Spijker et al., 2020; Laham and Gould, 2022). In Rett syndrome, excessive net maturation may prematurely restrict circuit flexibility (Carstens et al., 2021). In fragile X-related states, insufficient extracellular organization may impair interneuron maturation. In schizophrenia-related pathology, reduced PNN support may destabilize PV interneuron function and cortical timing (Siller and Broadie, 2012; Pantazopoulos and Berretta, 2016). In Alzheimer’s disease, altered proteoglycan composition and Reelin dysregulation may simultaneously affect toxic protein handling, synapse resilience, and compensatory plasticity (Goedert et al., 1996; Scholefield et al., 2003; Holmes et al., 2013; Liu et al., 2016; Suttkus et al., 2016; Crapser et al., 2020). Accordingly, ECM biology recurs across otherwise disparate neurological and psychiatric disorders because extracellular remodeling is a fundamental mechanism by which the nervous system balances preservation of established circuit architecture with the capacity for adaptive change.

## 6 ECM and PNNs in epilepsy

### 6.1 Epilepsy: from abnormal discharges to chronic network disease

Epilepsy is not adequately captured by the simple appearance of neurons firing “too much.” Clinically, the disorder is defined by a persistent predisposition to generate spontaneous seizures, but biologically it is better understood as a chronic disease of circuit organization, in which transient episodes of hypersynchronous activity become embedded within a progressively altered neural network (Zhu et al., 2022; Zhu et al., 2023; Zhu et al., 2024). This distinction between ictogenesis and epileptogenesis is fundamental. Ictogenesis concerns the immediate generation and spread of a seizure; epileptogenesis concerns the slower conversion of previously non-epileptic tissue into a seizure-generating substrate (Pitkänen and Engel, 2014). The latter process requires durable changes in neuronal connectivity, inhibitory restraint, glial behavior, vascular integrity, and extracellular signaling. In this framework, epilepsy is not merely an electrical disorder but a disorder of pathological tissue memory, in which abnormal activity is translated into lasting structural and molecular reconfiguration. For example, PNNs are markedly altered in the epileptic cortex. In cryptogenic epilepsy, aggrecan expression is severely reduced and its normal cortical gradient is lost, with residual PNNs mainly surrounding parvalbumin-positive interneurons. In focal cortical dysplasia type II, PNN organization is disrupted within the dysplastic core and perilesional cortex, with abnormal PNNs also forming around dysmorphic neurons. These findings indicate that loss and reorganization of PNNs are prominent pathological features of epilepsy and may contribute to impaired synaptic stability, inhibitory circuit function, and network plasticity (De Santis et al., 2026; see Table 1). The ECM is therefore not peripheral to the disease. It is one of the principal systems through which the brain stabilizes mature circuitry, limits excessive remodeling, and governs how experience or injury is converted into architecture (Dityatev and Fellin, 2008; McRae and Porter, 2012; Soleman et al., 2013).

**Table 1:** The characteristics of ECM and PNNs in epilepsy.

Feature	Diffuse/interstitial or perisynaptic ECM	Perineuronal nets (condensed ECM)	Interpretive relevance to epilepsy
Core molecular organization	HA; lecticans such as brevican/neurocan/versican/aggrecan to variable degrees; tenascins; glycoproteins; extracellular enzymes and signaling ligands.	HA backbone with concentrated lecticans, especially aggrecan, stabilized by HAPLN proteins and tenascin-R; characteristic CS-glycan patterns and bound ligands.	Seizures may alter scaffold abundance, glycan sulfation, proteolysis, or spatial condensation rather than changing all ECM molecules uniformly.
Cellular sources	Neurons, astrocytes, oligodendroglial-lineage cells and other glial/non-neuronal sources depending on molecule and state.	Both net-bearing neurons and surrounding glial cells can contribute components; cellular origin is molecule- and region-dependent (Carulli et al., 2006).	Neuronal synthesis; reactive glia can become major ECM/protease sources after injury.
Synaptic / receptor effects	Compartmentalize membrane receptor mobility and regulate receptor exchange, diffusion of signaling molecules and perisynaptic environment (Frischknecht et al., 2009).	Stabilizes a mature perisomatic/proximal-dendritic microdomain and can regulate the physiological state of PNN-bearing neurons.	Changes in diffuse ECM may affect excitatory receptor mobility even when a PNN is not directly involved.
PV interneuron relationship	Indirect/broad effects through extracellular and synaptic environment.	Strong association with many mature PV + fast-spiking interneurons; attenuation can reduce fast-spiking gain/excitability in some circuits (Balmer, 2016).	PNN loss can affect inhibitory timing, but effects are circuit- and manipulation-dependent and should not be generalized to all PV cells.
Biophysical functions	Strong relevance to extracellular-space volume, diffusion and local ionic environment; HA deficiency provides direct evidence (Arranz et al., 2014).	Highly polyanionic local microenvironment; proposed contributions to cation buffering and protection/stability.	Epilepsy mechanisms include both structural plasticity and immediate extracellular biophysics.
Plasticity	Constrains receptor mobility and structural/synaptic remodeling but remains dynamically remodelable.	Associated with critical-period closure and restriction of some adult plasticity; removal can reopen plasticity.	Increased plasticity can be adaptive or maladaptive depending on task, circuit and disease stage.
Interpretation of ChABC	ChABC cleaves CS chains in CSPG-rich ECM broadly.	PNNs are prominently attenuated, but ChABC is not intrinsically PNN-specific.	Avoid claiming a PNN-specific mechanism solely from ChABC.
Epilepsy examples	Has3 loss reduces ECS and promotes seizures; BBB/glial signaling and extracellular proteases modify diffuse ECM.	SE-associated aggrecan/HAPLN1/HAS3 loss; MMP-cleaved aggrecan around PV cells; human PNN abnormalities.	The disease phenotype may result from loss, accumulation, redistribution or altered sulfation, depending on model/stage/etiology.

Temporal lobe epilepsy (TLE) provides perhaps the clear experimental and clinical example of this principle. Following an initial precipitating insult such as status epilepticus, trauma, ischemia, infection, or blood–brain barrier (BBB) failure, limbic circuits may enter a latent interval during which overt spontaneous seizures are absent but the tissue is already being reworked (Devinsky et al., 2018; Zhu et al., 2023; Zhu et al., 2024). Neuronal loss, reactive gliosis, dendritic remodeling, mossy fiber sprouting, synaptic reorganization, inflammatory signaling, and extracellular matrix turnover all unfold during this period (Borowicz-Reutt and Czuczwar, 2020). By the time chronic spontaneous seizures emerge, the hippocampal formation is not simply “irritable”; it has been remodeled. Thus, the central mechanistic question is not only why excitation exceeds inhibition at a given moment, but how that imbalance becomes structurally consolidated. The answer cannot be fully framed at the level of ion channels or synaptic vesicles alone, because network persistence depends on whether the extracellular environment permits, channels, or constrains circuit rewiring. In this respect, ECM biology enters at the level of first principles: it defines whether the mature brain remains resistant to aberrant sprouting or becomes permissive to pathological reconnection (van Vliet et al., 2007; Dityatev and Fellin, 2008; Soleman et al., 2013).

A major difficulty in epilepsy research is that epileptic tissue is neither uniformly degenerative nor uniformly plastic. It is both at once (Wang and Fawcett, 2012; McRae and Porter, 2012). Certain neuronal populations, particularly inhibitory interneurons or selectively vulnerable pyramidal cells, may be lost or functionally weakened (Podgorny et al., 2025); elsewhere, recurrent excitatory circuits are formed, dendritic arbors are reshaped, and axons sprout into territories that were previously inaccessible (Nasrallah et al., 2022; McCrimmon et al., 2025). The chronic epileptic brain is therefore not well described by a binary “too much excitation, too little inhibition” formula, even though such imbalance is undeniably present. A better account asks how the diseased brain re-enters a state of unstable structural permissiveness while simultaneously attempting to preserve function. This question highlights fast-spiking PV-positive interneurons and the PNNs that surround many of them. PNNs emerge late in development, coincide with the closure of critical periods, and act as molecular correlates of circuit consolidation (Chelini et al., 2018). Their relevance to epilepsy is therefore conceptual as well as mechanistic: if seizures weaken, fragment, or recompose these nets, then pathological activity gains access to a mature circuit that has partially lost its extracellular brakes on reorganization (Wang and Fawcett, 2012; McRae and Porter, 2012).

This perspective also explains why non-neuronal elements have moved from the margins to the center of epilepsy research. Astrocytes, microglia, endothelial cells, leukocyte trafficking, extracellular proteases, and ECM molecules do not merely modulate an otherwise neuronal disease; together they constitute the environment in which seizures are amplified, constrained, or consolidated (Chaunsali et al., 2021; Gaillard et al., 2025). Janigro and Walker argued that purely neurocentric accounts leave major features of epilepsy unexplained, especially those involving BBB maintenance, glial regulation of the extracellular milieu, and transitions between apparently quiescent and seizure-prone states (Janigro and Walker, 2014). The neuro-glial-vascular-matrix unit is therefore a practical disease framework. Once BBB integrity is compromised, albumin and other blood-derived factors gain abnormal access to the parenchyma, glial signaling is altered, extracellular ionic and molecular homeostasis shifts, and matrix turnover may accelerate. Such changes do not merely accompany epileptogenesis; they help create the biochemical and structural conditions under which epileptiform circuits can persist (van Vliet et al., 2007; Janigro and Walker, 2014).

For these reasons, epilepsy should be viewed not as a transient disorder of firing alone, but as a chronic disorder of maladaptive circuit stabilization. The ECM is central to that learning process. It regulates synaptic boundary conditions, influences the permissiveness of the extracellular space, and helps determine whether post-injury remodeling remains compensatory or turns self-reinforcing and epileptogenic. This concept is especially powerful in TLE, but it is likely relevant more broadly across acquired epilepsies in which recurrent activity, inflammation, and structural reorganization converge. In that sense, ECM and PNN biology do not simply add molecular detail to classical seizure theory; they alter its conceptual center of gravity.

### 6.2 The role of ECM and PNNs is involved in plasticity and circuit alterations in epilepsy

In the healthy brain, plasticity allows circuits to be modified by experience, consolidated into memory, and restored after perturbation (Šimončičová et al., 2024). These processes are not purely intracellular. They depend on coordinated interactions among synapses, adhesion systems, extracellular proteases, glia, and ECM macromolecules. Dityatev and Schachner (2003) emphasized more than two decades ago that ECM molecules are active participants in synaptic plasticity rather than passive background material. Wright and colleagues likewise linked ECM reconfiguration to LTP, synaptogenesis, and memory consolidation (Wright et al., 2002). These formulations place the extracellular space within the machinery of enduring synaptic change. Plasticity is not executed solely at the membrane or in the cytoskeleton; it is negotiated across the cell surface and the surrounding matrix (Xu et al., 2025).

Epileptogenesis can therefore be understood, in part, as the pathological capture of physiological plasticity mechanisms by abnormal patterns of activity. Seizure engages many of the same molecular and structural programs that underlie adaptive remodeling, but they do so at pathological intensity, repetition, and spatial scale (Blondiaux et al., 2023). The result is not plasticity in the ordinary sense, but maladaptive plasticity. Dendritic spines are reshaped, glutamatergic signaling is altered, axons sprout, inhibitory networks are reorganized, and trophic signaling is rebalanced. None of these changes is merely epiphenomenal (Dityatev and Fellin, 2008; McRae and Porter, 2012; Soleman et al., 2013). Each can alter information flow, reduce seizure threshold, and stabilize recurrent pathways that favor future seizures. The ECM is central in this process because it determines whether structural change is physically and molecularly permitted. To say that epilepsy is associated with aberrant plasticity is therefore incomplete. The more precise claim is that epilepsy reflects a failure of the mature extracellular environment to keep plasticity spatially restricted, functionally integrated, and temporally self-limited (Kim et al., 2016; Xu et al., 2025).

The anatomical positioning alone makes PNNs highly relevant to epilepsy. Any persistent alteration in their composition or integrity has the potential to disproportionately affect inhibitory precision at the level of entire circuits (Wang and Fawcett, 2012; McRae and Porter, 2012; Soleman et al., 2013). In physiological conditions, PNNs are associated with the closure of critical periods, the stabilization of established synaptic relationships, and the restriction of exuberant structural remodeling (Fawcett et al., 2022; Miyata et al., 2012). That restriction is not incidental. It is part of the molecular logic by which mature circuits preserve reliability. If seizures disrupt PNN integrity, then the adult brain may be driven back toward a more permissive and immature extracellular state (McRae and Porter, 2012; Mueller-Buehl et al., 2023). Such a state may transiently allow compensatory adaptation after injury, but it may also lower the barriers that ordinarily prevent recurrent axonal sprouting, abnormal synapse formation, and destabilization of inhibitory microcircuits (Blondiaux et al., 2023). The same extracellular structures that limit plasticity in maturity may, when degraded, reopen a period of structural malleability that the injured or hyperactive network exploits in maladaptive ways (McRae and Porter, 2012; Wang and Fawcett, 2012).

Direct evidence for seizure-induced PNN remodeling was provided by McRae and colleagues in the pilocarpine model (McRae et al., 2012). They showed that the aggrecan component of hippocampal PNNs was reduced after status epilepticus, with a decrease evident during the subacute period and persisting into the chronic stage. Importantly, this loss was not limited to aggrecan staining itself. It was preceded by reductions in hyaluronan, HAS3, and HAPLN1, molecules required for stabilizing the relationship between aggrecan and hyaluronan. The SE-exposed hippocampus also showed PNNs with poor structural integrity (McRae and Porter, 2012; McRae et al., 2012). Rankin-Gee et al. (2015) subsequently detected an MMP-generated aggrecan neoepitope around PV interneurons after status epilepticus and persistent aggrecan proteolysis. Importantly, ChABC altered different components of PTZ seizure behavior in different directions, emphasizing that PNN attenuation is not equivalent to a simple monotonic increase in seizure severity. Broad metalloproteinase inhibition can prevent PNN breakdown, inhibitory-synapse reorganization and seizure development in a kindling paradigm (Pollock et al., 2014). Notably, increased C6S and C6S-enriched PNNs after kainate treatment, a reduced 4S/6S ratio, and greater kainate seizure susceptibility in C6ST-1 transgenic mice (Yutsudo and Kitagawa, 2015). This finding shows that seizure-related ECM pathology can involve a shift in glycan chemistry even when a net remains histologically detectable. It also argues against using WFA or total CSPG staining as a complete proxy for functional ECM state. In addition, HA-rich diffuse ECM can influence seizures through extracellular biophysics. Arranz et al. (2014) demonstrated spontaneous seizures/epileptiform activity in Has3-deficient mice, a selective ~40% reduction of extracellular-space volume in CA1 stratum pyramidale, and reversible control of epileptiform activity by osmotic manipulation of extracellular-space volume. These findings indicate that seizure-induced net attenuation is not a superficial histological artifact but reflects destabilization of multiple interacting structural components. Put differently, the net is not merely “less visible”; it is being molecularly disassembled. That disassembly occurs precisely during the window in which epileptogenic circuit reorganization is unfolding.

The functional implications of such PNN degradation are substantial. First, inhibitory interneurons may lose elements of the perisomatic extracellular scaffold that help maintain stable synapticn and mature firing properties (McRae and Porter, 2012). Second, principal-cell circuits may experience fewer extracellular constraints on structural remodeling, increasing the likelihood that repeated activity is consolidated into aberrant recurrent connectivity (Chaunsali et al., 2021; Gaillard et al., 2025). Third, the extracellular distribution of signaling molecules, proteases, and trophic factors may change as matrix architecture loosens. None of these consequences alone is sufficient to explain epilepsy, but together they define a coherent pathogenic logic: seizures weaken part of the extracellular apparatus that normally resists large-scale rewiring, thereby shifting the tissue toward a permissive state of maladaptive plasticity. In this sense, PNN loss should not be treated merely as a marker of injury. It is plausibly one of the mechanisms by which seizures are “remembered” by the network (Dityatev and Fellin, 2008; McRae and Porter, 2012).

At the same time, the literature does not support an overly simple equation of “PNN loss = epilepsy.” Recurrent seizures arise from interacting genetic, synaptic, inflammatory, and circuit-level processes. However, the ECM/PNN axis is unusually well positioned to contribute to seizure-prone network transformation (Arranz et al., 2014; Fawcett, 2015; Wiera and Mozrzymas, 2021; Fawcett et al., 2022). HA is a major determinant of extracellular space volume and ionic diffusion properties, and Has3 deficiency reduces brain extracellular space and is associated with altered neuronal activity and spontaneous seizures. PNNs, in turn, are tightly linked to the maturation and stability of PV interneurons, whose fast, temporally precise inhibition is essential for restraining hypersynchronous activity. Repeated seizures, inflammation, and extracellular protease activation could therefore convert physiological matrix remodeling into maladaptive reorganization by destabilizing inhibitory timing, altering receptor mobility, perturbing ion buffering, and enabling aberrant synaptic rewiring. In this framework, epilepsy can be viewed not only as a disorder of excitation–inhibition imbalance, but also as a disorder in which the extracellular governance of plasticity becomes progressively compromised. Notably, acute and subacute experimental epileptogenesis may involve local PNN attenuation, but chronic human epilepsy can display a more complex histopathological picture (McRae et al., 2012; Lehner et al., 2024). A recent study of human hippocampal tissue reported an age-dependent increase in PNN density in both controls and TLE patients, together with evidence compatible with precocious ECM aging in epilepsy; notably, higher hippocampal PNN density was associated with better memory performance in the patient cohort (Lehner et al., 2024). These data should not be read as disproving seizure-induced PNN degradation. Rather, they caution that PNN remodeling in epilepsy is likely stage-dependent, region-dependent, and cell-type-dependent. Early epileptogenesis may require focal net weakening to permit rewiring, whereas chronic epilepsy may also involve compensatory or maladaptive restabilization in selected territories (McRae et al., 2012; Yang et al., 2022). That interpretation remains inferential, but it accords more closely with the available evidence than any unidirectional model of persistent global PNN loss.

Proteolytic enzymes are the most likely executors of many of these matrix changes, and among them MMP-9 remains the dominant candidate (Bouquier et al., 2020). Under physiological conditions, MMP-9 is required for hippocampal late-phase LTP and hippocampus-dependent memory, with MMP-9 levels and proteolytic activity increasing after stimuli that induce L-LTP (Nagy et al., 2006). Wang and colleagues further demonstrated that extracellular proteolysis by MMP-9 drives persistent dendritic spine enlargement in coordination with LTP (Wang et al., 2008). In epilepsy models, MMP-9 has been linked to aberrant synaptogenesis, dendritic spine remodeling, trophic factor processing, and cell death (Takács et al., 2010; Vafadari et al., 2016). Wilczynski et al. established its importance in epileptogenesis (Wilczynski et al., 2008); Mizoguchi et al. showed that it promotes PTZ kindling by converting pro-BDNF to mature BDNF (Mizoguchi et al., 2011); and Kim et al. demonstrated that after pilocarpine-induced status epilepticus, MMP-9 expression and activity increased while β1-integrin levels decreased, supporting a role for MMP-9 in integrin-mediated hippocampal cell death (Kim et al., 2009). Notably, the pathogenic significance of MMP-9 in epilepsy was demonstrated directly by Wilczynski and colleagues, who identified it as a synaptic enzyme and a key factor in two animal models of TLE (Wilczynski et al., 2008). In their study, susceptibility to pentylenetetrazol (PTZ) kindling was reduced in MMP-9-deficient mice and increased in transgenic rats overexpressing MMP-9. Importantly, both MMP-9 protein levels and enzymatic activity are significantly elevated following seizures, whereas MMP-9 deficiency also diminished seizure-evoked dendritic spine pruning and decreased aberrant synaptogenesis after mossy fiber sprouting, indicating that the enzyme was influencing the structural rewriting of the circuit rather than simply marking tissue injury. In traumatic brain injury models, overexpression of MMP-9 increased the proportion of mice that developed spontaneous seizures, whereas genetic deletion of MMP-9 reduced seizure occurrence. In addition, MMP-9-overexpressing mice showed a significantly shorter latency to the first epileptiform spike after PTZ administration, while MMP-9 knockout mice exhibited the opposite pattern (Pijet et al., 2018). Human relevance is supported by Konopka et al. (2013), who found prominent MMP-9 upregulation in dysplastic surgical tissue, including abnormal neurons/balloon cells and synaptic compartments. These results show that a single extracellular protease can influence several dimensions of disease biology at once: synaptic remodeling, trophic signaling, and cell-survival pathways. Yet precisely because MMP-9 also participates in physiological plasticity, crude global inhibition is unlikely to be an ideal therapeutic strategy. The problem is not MMP-9 as such, but when, where, and on which substrates it acts.

A further nuance, often overlooked, is that extracellular proteolysis in epilepsy is not always equivalent to neurodestruction. Takács and colleagues reported increased MMP-9 activity in two seizure models that did not produce widespread neuronal death, arguing that MMP-9 induction may also participate in homeostatic synaptic plasticity (Takács et al., 2010). This suggests that the same enzyme can support adaptive remodeling under some circumstances and pathological remodeling under others. The critical variables are context, duration, localization, and substrate availability. A mature theory of epileptic plasticity must therefore resist the temptation to classify ECM proteolysis as uniformly beneficial or uniformly harmful. The question is not whether extracellular remodeling occurs, but whether the specific pattern of remodeling stabilizes healthy compensation or propagates network instability (Dityatev and Fellin, 2008).

Other extracellular proteolytic systems reinforce the same principle. Wu et al. showed that the tPA/plasmin cascade regulates seizure-induced mossy fiber outgrowth through proteoglycan processing, directly demonstrating that epileptic axonal reorganization depends on the modification of specific ECM substrates (Wu et al., 2000). This study demonstrated *in vivo* that seizure-associated axonal reorganization depends on selective editing of ECM molecules in the extracellular space. The sprouting axon is not acting alone. It grows within a microenvironment that has been proteolytically rewritten. Yuan et al. linked protease-specific brevican cleavage to synaptic loss in the dentate gyrus after kainate treatment, suggesting that the seizure-altered matrix is not merely dissolved but selectively edited (Yuan et al., 2002). Le and Friedman further demonstrated that MMP-7 regulates cleavage of proNGF and can be neuroprotective following kainic acid-induced seizures, illustrating that not all matrix proteases point in the same pathological direction (Le and Friedman, 2012). The implication is critical: the ECM should not be treated as a single substrate class, and proteolysis should not be treated as a single biological meaning. Different enzymes acting on different matrix molecules at different times are likely to produce sharply different outcomes—sprouting, destabilization, compensation, or neuroprotection.

This molecular specificity is crucial for interpreting the ECM literature in epilepsy. Early work documented seizure-related changes in tenascin-C, tenascin-R, neurocan, phosphacan, aggrecan, and related components (Perkins et al., 2017; Balashova et al., 2019). McRae and Porter emphasized that neurocan and phosphacan are prominent CSPGs in the developing hippocampus and participate in axonal pathfinding, neurite growth, synapse formation, and plasticity (McRae and Porter, 2012). Soleman and colleagues further underscored that ECM molecules can act not only as modulators but, in some disorders, as causal factors, and that secondary ECM alterations can decisively influence regeneration and plasticity after CNS insults (Soleman et al., 2013). The lesson is that “the PNN” should not be treated as a unitary object whose pathology can be inferred from a single stain. What changes in epilepsy may be the relative balance among lecticans, link proteins, glycoproteins, sulfation patterns, cleavage fragments, and rates of turnover. Histological attenuation, regional accumulation, or altered density may therefore reflect different molecular states rather than contradictory observations.

The inflammatory and vascular context further complicates this matrix biology. BBB leakage during epileptogenesis exposes the parenchyma to albumin and other blood-derived factors, while inflammatory cells and activated glia contribute additional proteases, cytokines, and reactive mediators (van Vliet et al., 2007). Artificial opening of the BBB increased seizures in the majority of animals studied (Huang et al., 2025). Prolonged BBB opening or cortical exposure to serum/albumin is sufficient to produce a delayed epileptiform focus (Seiffert et al., 2004). Subsequent work implicated albumin-TGF-β signaling as a pro-epileptogenic pathway, with Bar-Klein et al. (2014) demonstrating antiepileptogenic effects of TGF-β pathway suppression. Kim and colleagues further connected TGF-β signaling after neurological insults with inflammatory gene expression and reduced PNN association around inhibitory neurons (Kim et al., 2017). This is not a peripheral issue. It implies that epileptic remodeling unfolds in an extracellular environment that has become chemically abnormal: serum proteins, inflammatory mediators, and vascular signals gain access to the parenchyma, altering astrocytic behavior, ionic buffering, and matrix turnover. In the process, MMP-9 may bridge neuroinflammation and seizure development by acting on BBB components and inflammatory pathways (Bronisz and Kurkowska-Jastrzębska, 2016). In such a framework, ECM and PNN remodeling are embedded within a feed-forward neurovascular-inflammatory cascade: seizures induce proteolysis and barrier dysfunction; barrier dysfunction amplifies inflammation and extracellular disequilibrium; these changes in turn further destabilize the matrix and the circuit. Epileptogenesis then becomes not merely a neuronal loop, but a tissue-level loop involving neurons, glia, vessels, and matrix. This integrated view is more difficult than the classical synaptic model, but it is also more faithful to the pathology observed in chronic epilepsy.

Interestingly, not all epileptic ECM phenotypes are degradative. Patel and colleagues found *de novo* increased CSPG deposition in dentate gyrus and amygdala of mice that seized after Theiler’s virus infection (Patel et al., 2024). Region-specific aggrecan deletion reduced seizure burden and normalized intrinsic/synaptic hyperexcitability of dentate granule cells, providing causal evidence that pathological ECM accumulation can be pro-ictogenic.

In addition, these findings carry important therapeutic implications. In regeneration research, enzymatic digestion of CSPGs or pharmacological interference with ECM signaling has often been explored as a means of reopening plasticity (Miyata and Kitagawa, 2015). In epilepsy, however, the logic is considerably less straightforward. Simply loosening the matrix may facilitate maladaptive sprouting, reduce inhibitory stabilization, and intensify circuit susceptibility. Simply preserving or reinforcing the matrix may instead lock in pathological connectivity and blunt compensatory reorganization (McRae and Porter, 2012; Soleman et al., 2013; Bronisz and Kurkowska-Jastrzębska, 2016). The therapeutic challenge is therefore not to increase or decrease matrix plasticity in the abstract, but to normalize the spatiotemporal pattern of ECM remodeling. That goal will likely require stage-specific and substrate-specific approaches: one type of intervention during the latent phase, another after chronic networks is established; one strategy for interneuronal PNN integrity, another for diffuse interstitial proteolysis; one set of targets for inflammatory protease cascades, another for trophic factor processing. Unfortunately, the field is not yet at the point of translating such distinctions into antiepileptogenic therapies.

From a theoretical standpoint, the study of ECM and PNNs in epilepsy compels a revision of classical seizure theory. The decisive pathological lesion may not reside solely in ion channels, neurotransmitter receptors, or synaptic release machinery, but also in the extracellular rules that determine whether activity can be converted into durable circuit architecture (McRae et al., 2012; Wang and Fawcett, 2012; Lehner et al., 2024). PNNs are especially important because they sit at the border between plasticity and stability. When intact, they help preserve mature inhibitory microcircuits and restrict excessive reorganization. When selectively weakened, they may open a phase of heightened structural lability during which aberrant rewiring becomes possible. When chronically altered or regionally accumulated, they may contribute to a dysfunctional form of restabilization that preserves pathological network states rather than healthy ones. This tripartite framework—protective restraint, permissive degradation, maladaptive restabilization—captures the present literature more accurately than any simple model of net loss or net excess. It also explains why PNNs can be linked simultaneously to seizure susceptibility, chronic circuit pathology, and memory outcome.

In summary, the most convincing current view is that ECM and PNN remodeling are not accessory phenomena in epilepsy but integral components of disease evolution. Seizures recruit physiological plasticity pathways, including extracellular proteolysis and matrix-dependent synaptic remodeling. These pathways alter the permissiveness of the extracellular space, the stability of inhibitory microdomains, the extent of axonal sprouting, and the crosstalk among neurons, glia, and the vasculature. MMP-9, tPA/plasmin, brevican cleavage, BBB dysfunction, and persistent changes in PNN organization all converge on the same principle: epileptogenesis involves coordinated changes not only in neurons but also in the extracellular milieu that shapes their connectivity and excitability. The major unresolved question is no longer whether the ECM matters in epilepsy, but when, where, and in which molecular form ECM remodeling crosses the line from adaptive plasticity to pathological self-organization.

## References

[ref1] Alzarea S. I. (2026). Role of Perineuronal nets in neuronal plasticity and repair: molecular perspectives on psychiatric disorders. Curr. Neuropharmacol. 24:3021. doi: 10.2174/011570159X434753260323193021, 42163671

[ref2] Arranz A. M. Perkins K. L. Irie F. Lewis D. P. Hrabe J. Xiao F. . (2014). Hyaluronan deficiency due to Has3 knock-out causes altered neuronal activity and seizures via reduction in brain extracellular space. J. Neurosci. 34, 6164–6176. doi: 10.1523/JNEUROSCI.3458-13.2014, 24790187 PMC4004806

[ref3] Aspberg A. Miura R. Bourdoulous S. Shimonaka M. Heinegård D. Schachner M. . (1997). The C-type lectin domains of lecticans, a family of aggregating chondroitin sulfate proteoglycans, bind tenascin-R by protein-protein interactions independent of carbohydrate moiety. Proc. Natl. Acad. Sci. U. S. A. 94, 10116–10121. doi: 10.1073/pnas.94.19.10116, 9294172 PMC23322

[ref4] Auer S. Schicht M. Hoffmann L. Budday S. Frischknecht R. Blümcke I. . (2025). The role of Perineuronal nets in physiology and disease: insights from recent studies. Cells 14:321. doi: 10.3390/cells14050321, 40072050 PMC11898492

[ref5] Baidoe-Ansah D. Carceller H. Mirzapourdelavar H. Pérez-Rando M. Strackeljan L. Garcia-Vazquez B. . (2026). Depletion of Neurocan in the prefrontal cortex impairs temporal order recognition, cognitive flexibility and Perisomatic GABAergic innervation. Cell. Mol. Neurobiol. 46:103. doi: 10.1007/s10571-026-01762-2, 42260248 PMC13247103

[ref6] Baidoe-Ansah D. Mirzapourdelavar H. Aleshin S. Schott B. H. Seidenbecher C. Kaushik R. . (2025). Neurocan regulates axon initial segment organization and neuronal activity. Matrix Biol. 136, 22–35. doi: 10.1016/j.matbio.2025.01.001, 39788215

[ref7] Balashova A. Pershin V. Zaborskaya O. Tkachenko N. Mironov A. Guryev E. . (2019). Enzymatic digestion of Hyaluronan-based brain extracellular matrix in vivo can induce seizures in neonatal mice. Front. Neurosci. 13:1033. doi: 10.3389/fnins.2019.01033, 31632233 PMC6779145

[ref8] Balmer T. S. (2016). Perineuronal nets enhance the excitability of fast-spiking neurons. eNeuro 3:16. doi: 10.1523/ENEURO.0112-16.2016, 27570824 PMC4987413

[ref9] Banerjee S. B. Gutzeit V. A. Baman J. Aoued H. S. Doshi N. K. Liu R. C. . (2017). Perineuronal nets in the adult sensory cortex are necessary for fear learning. Neuron 95, 169–179.e3. doi: 10.1016/j.neuron.2017.06.007, 28648500 PMC5548423

[ref10] Bar-Klein G. Cacheaux L. P. Kamintsky L. Prager O. Weissberg I. Schoknecht K. . (2014). Losartan prevents acquired epilepsy via TGF-β signaling suppression. Ann. Neurol. 75, 864–875. doi: 10.1002/ana.24147, 24659129 PMC4077937

[ref11] Beffert U. Weeber E. J. Durudas A. Qiu S. Masiulis I. Sweatt J. D. . (2005). Modulation of synaptic plasticity and memory by Reelin involves differential splicing of the lipoprotein receptor Apoer2. Neuron 47, 567–579. doi: 10.1016/j.neuron.2005.07.007, 16102539

[ref12] Berretta S. (2012). Extracellular matrix abnormalities in schizophrenia. Neuropharmacology 62, 1584–1597. doi: 10.1016/j.neuropharm.2011.08.010, 21856318 PMC3234338

[ref13] Beurdeley M. Spatazza J. Lee H. H. Lee H. H. C. Sugiyama S. Bernard C. . (2012). Otx2 binding to perineuronal nets persistently regulates plasticity in the mature visual cortex. J. Neurosci. 32, 9429–9437. doi: 10.1523/JNEUROSCI.0394-12.2012, 22764251 PMC3419577

[ref14] Bitanihirwe B. K. Woo T. U. (2014). Perineuronal nets and schizophrenia: the importance of neuronal coatings. Neurosci. Biobehav. Rev. 45, 85–99. doi: 10.1016/j.neubiorev.2014.03.018, 24709070 PMC4447499

[ref15] Blondiaux A. Jia S. Annamneedi A. Çalışkan G. Nebel J. Montenegro-Venegas C. . (2023). Linking epileptic phenotypes and neural extracellular matrix remodeling signatures in mouse models of epilepsy. Neurobiol. Dis. 188:106324. doi: 10.1016/j.nbd.2023.106324, 37838005

[ref16] Borowicz-Reutt K. K. Czuczwar S. J. (2020). Role of oxidative stress in epileptogenesis and potential implications for therapy. Pharmacol. Rep. 72, 1218–1226. doi: 10.1007/s43440-020-00143-w, 32865811 PMC7550371

[ref17] Boudkkazi S. Schwenk J. Nakaya N. Brechet A. Kollewe A. Harada H. . (2023). A Noelin-organized extracellular network of proteins required for constitutive and context-dependent anchoring of AMPA-receptors. Neuron 111, 2544–2556.e9. doi: 10.1016/j.neuron.2023.07.013, 37591201 PMC10441612

[ref18] Bouquier N. Girard B. Aparicio Arias J. Fagni L. Bertaso F. Perroy J. (2020). Gelatinase biosensor reports cellular remodeling during Epileptogenesis. Front. Synaptic Neurosci. 12:15. doi: 10.3389/fnsyn.2020.00015, 32372941 PMC7186352

[ref19] Bronisz E. Kurkowska-Jastrzębska I. (2016). Matrix metalloproteinase 9 in epilepsy: the role of Neuroinflammation in seizure development. Mediat. Inflamm. 2016, 1–14. doi: 10.1155/2016/7369020, 28104930 PMC5220508

[ref21] Brückner G. Szeöke S. Pavlica S. Grosche J. Kacza J. (2006). Axon initial segment ensheathed by extracellular matrix in perineuronal nets. Neuroscience 138, 365–375. doi: 10.1016/j.neuroscience.2005.11.068, 16427210

[ref22] Bukalo O. Schachner M. Dityatev A. (2001). Modification of extracellular matrix by enzymatic removal of chondroitin sulfate and by lack of tenascin-R differentially affects several forms of synaptic plasticity in the hippocampus. Neuroscience 104, 359–369. doi: 10.1016/S0306-4522(01)00082-3, 11377840

[ref23] Cabungcal J. H. Steullet P. Morishita H. Kraftsik R. Cuenod M. Hensch T. K. . (2013). Perineuronal nets protect fast-spiking interneurons against oxidative stress. Proc. Natl. Acad. Sci. U. S. A. 110, 9130–9135. doi: 10.1073/pnas.1300454110, 23671099 PMC3670388

[ref24] Cangalaya C. Sun W. Stoyanov S. Dunay I. R. Dityatev A. (2024). Integrity of neural extracellular matrix is required for microglia-mediated synaptic remodeling. Glia 72, 1874–1892. doi: 10.1002/glia.24588, 38946065

[ref25] Cardin J. A. Carlén M. Meletis K. Knoblich U. Zhang F. Deisseroth K. . (2009). Driving fast-spiking cells induces gamma rhythm and controls sensory responses. Nature 459, 663–667. doi: 10.1038/nature08002, 19396156 PMC3655711

[ref26] Carstens K. E. Lustberg D. J. Shaughnessy E. K. McCann K. E. Alexander G. M. Dudek S. M. (2021). Perineuronal net degradation rescues CA2 plasticity in a mouse model of Rett syndrome. J. Clin. Invest. 131:e137221. doi: 10.1172/JCI137221, 34228646 PMC8363283

[ref27] Carstens K. E. Phillips M. L. Pozzo-Miller L. Weinberg R. J. Dudek S. M. (2016). Perineuronal nets suppress plasticity of excitatory synapses on CA2 pyramidal neurons. J. Neurosci. 36, 6312–6320. doi: 10.1523/JNEUROSCI.0245-16.2016, 27277807 PMC4899529

[ref28] Carulli D. Pizzorusso T. Kwok J. C. Kwok J. C. F. Putignano E. Poli A. . (2010). Animals lacking link protein have attenuated perineuronal nets and persistent plasticity. Brain 133, 2331–2347. doi: 10.1093/brain/awq145, 20566484

[ref29] Carulli D. Rhodes K. E. Brown D. J. Bonnert T. P. Pollack S. J. Oliver K. . (2006). Composition of perineuronal nets in the adult rat cerebellum and the cellular origin of their components. J. Comp. Neurol. 494, 559–577. doi: 10.1002/cne.20822, 16374793

[ref30] Celio M. R. Spreafico R. De Biasi S. Vitellaro-Zuccarello L. (1998). Perineuronal nets: past and present. Trends Neurosci. 21, 510–515. doi: 10.1016/S0166-2236(98)01298-3, 9881847

[ref31] Chang M. C. Park J. M. Pelkey K. A. Grabenstatter H. L. Xu D. Linden D. J. . (2010). Narp regulates homeostatic scaling of excitatory synapses on parvalbumin-expressing interneurons. Nat. Neurosci. 13, 1090–1097. doi: 10.1038/nn.2621, 20729843 PMC2949072

[ref32] Chaunsali L. Tewari B. P. Sontheimer H. (2021). Perineuronal net dynamics in the pathophysiology of epilepsy. Epilepsy Curr. 21, 273–281. doi: 10.1177/15357597211018688, 34690566 PMC8512927

[ref33] Chelini G. Pantazopoulos H. Durning P. Berretta S. (2018). The tetrapartite synapse: a key concept in the pathophysiology of schizophrenia. Eur. Psychiatry 50, 60–69. doi: 10.1016/j.eurpsy.2018.02.003, 29503098 PMC5963512

[ref34] Christensen A. C. Lensjø K. K. Lepperød M. E. Dragly S. A. Sutterud H. Blackstad J. S. . (2021). Perineuronal nets stabilize the grid cell network. Nat. Commun. 12:253. doi: 10.1038/s41467-020-20241-w, 33431847 PMC7801665

[ref35] Cingolani L. A. Vitale C. Dityatev A. (2019). Intra- and extracellular pillars of a unifying framework for homeostatic plasticity: a crosstalk between metabotropic receptors and extracellular matrix. Front. Cell. Neurosci. 13:513. doi: 10.3389/fncel.2019.00513, 31803023 PMC6877475

[ref36] Crapser J. D. Spangenberg E. E. Barahona R. A. Arreola M. A. Hohsfield L. A. Green K. N. (2020). Microglia facilitate loss of perineuronal nets in the Alzheimer’s disease brain. EBioMedicine 58:102919. doi: 10.1016/j.ebiom.2020.102919, 32745992 PMC7399129

[ref37] Dankovich T. M. Kaushik R. Olsthoorn L. H. M. Petersen G. C. Giro P. E. Kluever V. . (2021). Extracellular matrix remodeling through endocytosis and resurfacing of tenascin-R. Nat. Commun. 12:7129. doi: 10.1038/s41467-021-27462-7, 34880248 PMC8654841

[ref38] De Luca C. Papa M. (2016). Looking inside the matrix: Perineuronal nets in plasticity, maladaptive plasticity and neurological disorders. Neurochem. Res. 41, 1507–1515. doi: 10.1007/s11064-016-1876-2, 26935742

[ref39] De Luca C. Papa M. (2017). Matrix metalloproteinases, neural extracellular matrix, and central nervous system pathology. Prog. Mol. Biol. Transl. Sci. 148, 167–202. doi: 10.1016/bs.pmbts.2017.04.002, 28662822

[ref40] De Santis D. Cagnoli C. Biancheri D. Pastori C. Medici V. Calatozzolo C. . (2026). Perineuronal net abnormalities in epileptic human tissue. Epilepsia 1:368. doi: 10.1002/epi.70368, 42377317

[ref41] Deepa S. S. Carulli D. Galtrey C. Rhodes K. Fukuda J. Mikami T. . (2006). Composition of perineuronal net extracellular matrix in rat brain: a different disaccharide composition for the net-associated proteoglycans. J. Biol. Chem. 281, 17789–17800. doi: 10.1074/jbc.M600544200, 16644727

[ref42] Devinsky O. Vezzani A. O’Brien T. J. Jette N. Scheffer I. E. de Curtis M. . (2018). Epilepsy. Nat. Rev. Dis. Primers 4:18024. doi: 10.1038/nrdp.2018.24, 29722352

[ref43] Dityatev A. Brückner G. Dityateva G. Grosche J. Kleene R. Schachner M. (2007). Activity-dependent formation and functions of chondroitin sulfate-rich extracellular matrix of perineuronal nets. Dev. Neurobiol. 67, 570–588. doi: 10.1002/dneu.20361, 17443809

[ref44] Dityatev A. Fellin T. (2008). Extracellular matrix in plasticity and epileptogenesis. Neuron Glia Biol. 4, 235–247. doi: 10.1017/S1740925X09000118, 19497143

[ref45] Dityatev A. Rusakov D. A. (2011). Molecular signals of plasticity at the tetrapartite synapse. Curr. Opin. Neurobiol. 21, 353–359. doi: 10.1016/j.conb.2010.12.006, 21277196 PMC3368316

[ref46] Dityatev A. Schachner M. (2003). Extracellular matrix molecules and synaptic plasticity. Nat. Rev. Neurosci. 4, 456–468. doi: 10.1038/nrn1115, 12778118

[ref9001] Dityatev A. Wehrle-Haller B. Pitkänen A. (2014). Preface. Brain extracellular matrix in health and disease. Prog Brain Res. 214, xiii–xvii., 25410369 10.1016/B978-0-444-63486-3.09998-9

[ref47] Downs M. Zaia J. Sethi M. K. (2023). Mass spectrometry methods for analysis of extracellular matrix components in neurological diseases. Mass Spectrom. Rev. 42, 1848–1875. doi: 10.1002/mas.21792, 35719114 PMC9763553

[ref48] Duncan J. A. Foster R. Kwok J. C. F. (2019). The potential of memory enhancement through modulation of perineuronal nets. Br. J. Pharmacol. 176, 3611–3621. doi: 10.1111/bph.14672, 30924524 PMC6715611

[ref49] Duncan B. W. Murphy K. E. Maness P. F. (2021). Molecular mechanisms of L1 and NCAM adhesion molecules in synaptic pruning, plasticity, and stabilization. Front. Cell Dev. Biol. 9:625340. doi: 10.3389/fcell.2021.625340, 33585481 PMC7876315

[ref50] Durakoglugil M. S. Chen Y. White C. L. Kavalali E. T. Herz J. (2009). Reelin signaling antagonizes beta-amyloid at the synapse. Proc. Natl. Acad. Sci. U. S. A. 106, 15938–15943. doi: 10.1073/pnas.0908176106, 19805234 PMC2747222

[ref51] Dzyubenko E. Gottschling C. Faissner A. (2016). Neuron-glia interactions in neural plasticity: contributions of neural extracellular matrix and Perineuronal nets. Neural Plast. 2016, 1–14. doi: 10.1155/2016/5214961, 26881114 PMC4736403

[ref52] Dzyubenko E. Hermann D. M. (2023). Role of glia and extracellular matrix in controlling neuroplasticity in the central nervous system. Semin. Immunopathol. 45, 377–387. doi: 10.1007/s00281-023-00989-1, 37052711 PMC10279577

[ref53] Dzyubenko E. Hermann D. M. (2025). Neuroglia and extracellular matrix molecules. Handb. Clin. Neurol. 209, 197–211. doi: 10.1016/B978-0-443-19104-6.00010-3, 40122625

[ref54] Evers M. R. Salmen B. Bukalo O. Rollenhagen A. Bösl M. R. Morellini F. . (2002). Impairment of L-type Ca2+ channel-dependent forms of hippocampal synaptic plasticity in mice deficient in the extracellular matrix glycoprotein tenascin-C. J. Neurosci. 22, 7177–7194. doi: 10.1523/JNEUROSCI.22-16-07177.2002, 12177213 PMC6757873

[ref55] Fawcett J. W. (2015). The extracellular matrix in plasticity and regeneration after CNS injury and neurodegenerative disease. Prog. Brain Res. 218, 213–226. doi: 10.1016/bs.pbr.2015.02.001, 25890139

[ref56] Fawcett J. W. Fyhn M. Jendelova P. Kwok J. C. F. Ruzicka J. Sorg B. A. (2022). The extracellular matrix and perineuronal nets in memory. Mol. Psychiatry 27, 3192–3203. doi: 10.1038/s41380-022-01634-3, 35760878 PMC9708575

[ref57] Fawcett J. W. Kwok J. C. F. (2022). Proteoglycan Sulphation in the function of the mature central nervous system. Front. Integr. Neurosci. 16:895493. doi: 10.3389/fnint.2022.895493, 35712345 PMC9195417

[ref58] Fawcett J. W. Oohashi T. Pizzorusso T. (2019). The roles of perineuronal nets and the perinodal extracellular matrix in neuronal function. Nat. Rev. Neurosci. 20, 451–465. doi: 10.1038/s41583-019-0196-3, 31263252

[ref59] Frischknecht R. Chang K. J. Rasband M. N. Seidenbecher C. I. (2014). Neural ECM molecules in axonal and synaptic homeostatic plasticity. Prog. Brain Res. 214, 81–100. doi: 10.1016/B978-0-444-63486-3.00004-9, 25410354

[ref60] Frischknecht R. Heine M. Perrais D. Seidenbecher C. I. Choquet D. Gundelfinger E. D. (2009). Brain extracellular matrix affects AMPA receptor lateral mobility and short-term synaptic plasticity. Nat. Neurosci. 12, 897–904. doi: 10.1038/nn.2338, 19483686

[ref61] Gaillard J. R. Gupta G. Mefford H. C. O’Brien L. M. Shellhaas R. A. Dang L. T. (2025). The association between sleep phenotypes and epilepsy genes. Neurol. Genet. 11:e200269. doi: 10.1212/NXG.0000000000200269, 40661862 PMC12258815

[ref62] Galtrey C. M. Fawcett J. W. (2007). The role of chondroitin sulfate proteoglycans in regeneration and plasticity in the central nervous system. Brain Res. Rev. 54, 1–18. doi: 10.1016/j.brainresrev.2006.09.006, 17222456

[ref63] Galtrey C. M. Kwok J. C. Carulli D. Kwok J. C. F. Rhodes K. E. Fawcett J. W. (2008). Distribution and synthesis of extracellular matrix proteoglycans, hyaluronan, link proteins and tenascin-R in the rat spinal cord. Eur. J. Neurosci. 27, 1373–1390. doi: 10.1111/j.1460-9568.2008.06108.x 18364019

[ref64] Garcion E. Faissner A. ffrench-Constant C. (2001). Knockout mice reveal a contribution of the extracellular matrix molecule tenascin-C to neural precursor proliferation and migration. Development 128, 2485–2496. doi: 10.1242/dev.128.13.2485, 11493565

[ref65] Goedert M. Jakes R. Spillantini M. G. Hasegawa M. Smith M. J. Crowther R. A. (1996). Assembly of microtubule-associated protein tau into Alzheimer-like filaments induced by sulphated glycosaminoglycans. Nature 383, 550–553. doi: 10.1038/383550a0, 8849730

[ref66] Gogolla N. Caroni P. Lüthi A. Herry C. (2009). Perineuronal nets protect fear memories from erasure. Science 325, 1258–1261. doi: 10.1126/science.1174146, 19729657

[ref67] Grødem S. Thompson E. H. Røe M. B. Vatne G. H. Nymoen Nystuen I. Buccino A. . (2025). Differential impacts of germline and adult aggrecan knockout in PV+ neurons on perineuronal nets and PV+ neuronal function. Mol. Psychiatry 30, 2907–2921. doi: 10.1038/s41380-025-02894-5, 39837996 PMC12185343

[ref68] Gu W. L. Fu S. L. Wang Y. X. Li Y. Lü H. Z. Xu X. M. . (2009). Chondroitin sulfate proteoglycans regulate the growth, differentiation and migration of multipotent neural precursor cells through the integrin signaling pathway. BMC Neurosci. 10:128. doi: 10.1186/1471-2202-10-128, 19845964 PMC2773784

[ref69] Happel M. F. Niekisch H. Castiblanco Rivera L. L. Happel M. F. K. Ohl F. W. Deliano M. . (2014). Enhanced cognitive flexibility in reversal learning induced by removal of the extracellular matrix in auditory cortex. Proc. Natl. Acad. Sci. USA 111, 2800–2805. doi: 10.1073/pnas.1310272111, 24550310 PMC3932855

[ref70] Härtig W. Brauer K. Bigl V. . (1994). Chondroitin sulfate proteoglycan-immunoreactivity of lectin-labeled perineuronal nets around parvalbumin-containing neurons. Brain Res. 635, 307–311. doi: 10.1016/0006-8993(94)91452-4, 8173967

[ref71] Heck N. Garwood J. Loeffler J. P. Larmet Y. Faissner A. (2004). Differential upregulation of extracellular matrix molecules associated with the appearance of granule cell dispersion and mossy fiber sprouting during epileptogenesis in a murine model of temporal lobe epilepsy. Neuroscience 129, 309–324. doi: 10.1016/j.neuroscience.2004.06.078, 15501589

[ref72] Hendrickson A. S. Francis K. L. Kumar A. le J. P. Scarlett J. M. Keene C. D. . (2024). Assessing translational applicability of perineuronal net dysfunction in Alzheimer’s disease across species. Front. Neurosci. 18:1396101. doi: 10.3389/fnins.2024.1396101, 38745932 PMC11091253

[ref73] Hodebourg R. Scofield M. D. Kalivas P. W. Kuhn B. N. (2025). Nonneuronal contributions to synaptic function. Neuron 113, 2399–2415. doi: 10.1016/j.neuron.2025.04.004, 40311612

[ref74] Holmes B. B. DeVos S. L. Kfoury N. Li M. Jacks R. Yanamandra K. . (2013). Heparan sulfate proteoglycans mediate internalization and propagation of specific proteopathic seeds. Proc. Natl. Acad. Sci. U. S. A. 110, E3138–E3147. doi: 10.1073/pnas.1301440110, 23898162 PMC3746848

[ref75] Huang N. Huang Y. Deng Z. Qi S. Zhang W. Liu Y. . (2025). Blood-brain barrier dysfunction in epilepsy: mechanisms, therapeutic strategies and future orientation (review). Int. J. Mol. Med. 56, 1–23. doi: 10.3892/ijmm.2025.5577, 40613229 PMC12270381

[ref76] Hylin M. J. Orsi S. A. Moore A. N. Dash P. K. (2013). Disruption of the perineuronal net in the hippocampus or medial prefrontal cortex impairs fear conditioning. Learn. Mem. 20, 267–273. doi: 10.1101/lm.030197.112, 23592037 PMC3630486

[ref77] Janigro D. Walker M. C. (2014). What non-neuronal mechanisms should be studied to understand epileptic seizures? Adv. Exp. Med. Biol. 813, 253–264. doi: 10.1007/978-94-017-8914-1_20, 25012382 PMC4842021

[ref78] Khoo G. H. Lin Y. T. Tsai T. C. Hsu K. S. (2019). Perineuronal nets restrict the induction of long-term depression in the mouse hippocampal CA1 region. Mol. Neurobiol. 56, 6436–6450. doi: 10.1007/s12035-019-1526-1, 30826967

[ref79] Kim G. W. Kim H. J. Cho K. J. Kim H. W. Cho Y. J. Lee B. I. (2009). The role of MMP-9 in integrin-mediated hippocampal cell death after pilocarpine-induced status epilepticus. Neurobiol. Dis. 36, 169–180. doi: 10.1016/j.nbd.2009.07.008, 19631748

[ref80] Kim S. Y. Porter B. E. Friedman A. Kaufer D. (2016). A potential role for glia-derived extracellular matrix remodeling in postinjury epilepsy. J. Neurosci. Res. 94, 794–803. doi: 10.1002/jnr.23758, 27265805

[ref81] Kim S. Y. Senatorov V. V. Jr. Morrissey C. S. Lippmann K. Vazquez O. Milikovsky D. Z. . (2017). TGFβ signaling is associated with changes in inflammatory gene expression and perineuronal net degradation around inhibitory neurons following various neurological insults. Sci. Rep. 7:7711. doi: 10.1038/s41598-017-07394-3, 28794441 PMC5550510

[ref82] Knapska E. Lioudyno V. Kiryk A. Mikosz M. Górkiewicz T. Michaluk P. . (2013). Reward learning requires activity of matrix metalloproteinase-9 in the central amygdala. J. Neurosci. 33, 14591–14600. doi: 10.1523/JNEUROSCI.5239-12.2013, 24005309 PMC6618374

[ref83] Kochlamazashvili G. Henneberger C. Bukalo O. Dvoretskova E. Senkov O. Lievens P. M. J. . (2010). The extracellular matrix molecule hyaluronic acid regulates hippocampal synaptic plasticity by modulating postsynaptic L-type ca(2+) channels. Neuron 67, 116–128. doi: 10.1016/j.neuron.2010.05.030, 20624596 PMC3378029

[ref84] Konopka A. Grajkowska W. Ziemiańska K. Roszkowski M. Daszkiewicz P. Rysz A. . (2013). Matrix metalloproteinase-9 (MMP-9) in human intractable epilepsy caused by focal cortical dysplasia. Epilepsy Res. 104, 45–58. doi: 10.1016/j.eplepsyres.2012.09.018, 23182966

[ref85] Kwok J. C. F. Dityatev A. (2026). Extracellular matrix remodelling in neurological diseases. Nat. Rev. Neurol. 22, 366–384. doi: 10.1038/s41582-026-01209-8, 42020548

[ref86] Laham B. J. Gould E. (2022). How stress influences the dynamic plasticity of the brain’s extracellular matrix. Front. Cell. Neurosci. 15:814287. doi: 10.3389/fncel.2021.814287, 35145379 PMC8821883

[ref87] Lavekar S. S. Patel M. D. Montalvo-Parra M. D. Krencik R. (2023). Asteroid impact: the potential of astrocytes to modulate human neural networks within organoids. Front. Neurosci. 17:1305921. doi: 10.3389/fnins.2023.1305921, 38075269 PMC10702564

[ref88] Le A. P. Friedman W. J. (2012). Matrix metalloproteinase-7 regulates cleavage of pro-nerve growth factor and is neuroprotective following kainic acid-induced seizures. J. Neurosci. 32, 703–712. doi: 10.1523/JNEUROSCI.4128-11.2012, 22238106 PMC3874860

[ref89] Lehner A. Hoffmann L. Rampp S. Coras R. Paulsen F. Frischknecht R. . (2024). Age-dependent increase of perineuronal nets in the human hippocampus and precocious aging in epilepsy. Epilepsia Open 9, 1372–1381. doi: 10.1002/epi4.12963, 38845524 PMC11296138

[ref90] Lensjø K. K. Lepperød M. E. Dick G. Hafting T. Fyhn M. (2017). Removal of Perineuronal nets unlocks juvenile plasticity through network mechanisms of decreased inhibition and increased gamma activity. J. Neurosci. 37, 1269–1283. doi: 10.1523/JNEUROSCI.2504-16.2016, 28039374 PMC6596863

[ref91] Lepelletier F. X. Mann D. M. Robinson A. C. Lepelletier F.‐. X. Mann D. M. A. Pinteaux E. . (2017). Early changes in extracellular matrix in Alzheimer’s disease. Neuropathol. Appl. Neurobiol. 43, 167–182. doi: 10.1111/nan.12295, 26544797

[ref92] Li X. Li N. Zhao P. Ren D. Luo B. Zhou T. (2025). Perineuronal nets: from structure to neurological disorders. Curr. Med. Chem. 32, 1685–1701. doi: 10.2174/0109298673258290231009111633, 37946343

[ref93] Liu L. Zhang Y. Men S. Li X. Hou S. T. Ju J. (2023). Elimination of perineuronal nets in CA1 disrupts GABA release and long-term contextual fear memory retention. Hippocampus 33, 862–871. doi: 10.1002/hipo.23503, 36709413

[ref94] Liu C. C. Zhao N. Yamaguchi Y. Cirrito J. R. Kanekiyo T. Holtzman D. M. . (2016). Neuronal heparan sulfates promote amyloid pathology by modulating brain amyloid-β clearance and aggregation in Alzheimer’s disease. Sci. Transl. Med. 8:332ra44. doi: 10.1126/scitranslmed.aad3650, 27030596 PMC5512541

[ref95] Lugara E. Van Vliet E. A. Romagnolo A. Staley K. Lillis K. P. Dulla C. . (2026). WONOEP XVII appraisal: the role of the extracellular matrix in epilepsy. Epilepsia 67, 3894–3908. doi: 10.1002/epi.70278, 42096301 PMC13525558

[ref96] Maeda N. Ishii M. Nishimura K. Kamimura K. (2011). Functions of chondroitin sulfate and heparan sulfate in the developing brain. Neurochem. Res. 36, 1228–1240. doi: 10.1007/s11064-010-0324-y, 21110089

[ref97] Marco A. (2022). Activity-dependent remodeling of genome architecture in engram cells facilitates memory formation and recall. Neural Regen. Res. 17, 991–993. doi: 10.4103/1673-5374.324834, 34558515 PMC8552845

[ref98] McCrimmon C. M. Toker D. Pahos M. Cao Q. Lozano K. Lin J. J. . (2025). Cortical versus hippocampal network dysfunction in a human brain assembloid model of epilepsy and intellectual disability. Cell Rep. 44:116217. doi: 10.1016/j.celrep.2025.116217, 40925365 PMC12674601

[ref99] McDonald A. J. Hamilton P. G. Barnstable C. J. (2018). Perineuronal nets labeled by monoclonal antibody VC1.1 ensheath interneurons expressing parvalbumin and calbindin in the rat amygdala. Brain Struct. Funct. 223, 1133–1148. doi: 10.1007/s00429-017-1542-8, 29094304 PMC5871560

[ref100] McRae P. A. Baranov E. Rogers S. L. Porter B. E. (2012). Persistent decrease in multiple components of the perineuronal net following status epilepticus. Eur. J. Neurosci. 36, 3471–3482. doi: 10.1111/j.1460-9568.2012.08268.x, 22934955 PMC4058987

[ref101] McRae P. A. Porter B. E. (2012). The perineuronal net component of the extracellular matrix in plasticity and epilepsy. Neurochem. Int. 61, 963–972. doi: 10.1016/j.neuint.2012.08.007, 22954428 PMC3930202

[ref102] Melrose J. Hayes A. J. Bix G. (2021). The CNS/PNS extracellular matrix provides instructive guidance cues to neural cells and Neuroregulatory proteins in neural development and repair. Int. J. Mol. Sci. 22:5583. doi: 10.3390/ijms22115583, 34070424 PMC8197505

[ref103] Mienaltowski M. J. Haddox N. L. Beall J. M. Pechanec M. Y. (2021). Basic structure, physiology, and biochemistry of connective tissues and extracellular matrix collagens. Adv. Exp. Med. Biol. 1348, 5–43. doi: 10.1007/978-3-030-80614-9_2, 34807414

[ref104] Miyata S. Kitagawa H. (2015). Mechanisms for modulation of neural plasticity and axon regeneration by chondroitin sulphate. J. Biochem. 157, 13–22. doi: 10.1093/jb/mvu067, 25381371

[ref105] Miyata S. Kitagawa H. (2017). Formation and remodeling of the brain extracellular matrix in neural plasticity: roles of chondroitin sulfate and hyaluronan. Biochim. Biophys. Acta Gen. Subj. 1861, 2420–2434. doi: 10.1016/j.bbagen.2017.06.010, 28625420

[ref106] Miyata S. Komatsu Y. Yoshimura Y. Taya C. Kitagawa H. (2012). Persistent cortical plasticity by upregulation of chondroitin 6-sulfation. Nat. Neurosci. 15, 414–422. doi: 10.1038/nn.3023, 22246436

[ref107] Mizoguchi H. Nakade J. Tachibana M. Ibi D. Someya E. Koike H. . (2011). Matrix metalloproteinase-9 contributes to kindled seizure development in pentylenetetrazole-treated mice by converting pro-BDNF to mature BDNF in the hippocampus. J. Neurosci. 31, 12963–12971. doi: 10.1523/JNEUROSCI.3118-11.2011, 21900575 PMC6623408

[ref108] Morawski M. Dityatev A. Hartlage-Rübsamen M. Blosa M. Holzer M. Flach K. . (2014). Tenascin-R promotes assembly of the extracellular matrix of perineuronal nets via clustering of aggrecan. Philos. Trans. R. Soc. Lond. Ser. B Biol. Sci. 369:20140046. doi: 10.1098/rstb.2014.0046, 25225104 PMC4173296

[ref109] Mueller-Buehl C. Wegrzyn D. Bauch J. Faissner A. (2023). Regulation of the E/I-balance by the neural matrisome. Front. Mol. Neurosci. 16:1102334. doi: 10.3389/fnmol.2023.1102334, 37143468 PMC10151766

[ref110] Nagy V. Bozdagi O. Matynia A. Balcerzyk M. Okulski P. Dzwonek J. . (2006). Matrix metalloproteinase-9 is required for hippocampal late-phase long-term potentiation and memory. J. Neurosci. 26, 1923–1934. doi: 10.1523/JNEUROSCI.4359-05.2006, 16481424 PMC4428329

[ref111] Nakajo H. Cao R. Mula S. A. McKetney J. Silva N. J. Li K. H. . (2026). Extracellular matrix proteolysis maintains synapse plasticity during brain development. Nat. Neurosci. 29, 567–580. doi: 10.1038/s41593-025-02153-4, 41430471 PMC12971489

[ref112] Nasrallah K. Frechou M. A. Yoon Y. J. Persaud S. Gonçalves J. T. Castillo P. E. (2022). Seizure-induced strengthening of a recurrent excitatory circuit in the dentate gyrus is proconvulsant. Proc. Natl. Acad. Sci. U. S. A. 119:e2201151119. doi: 10.1073/pnas.2201151119, 35930664 PMC9371717

[ref113] Nguyen P. T. Dorman L. C. Pan S. Vainchtein I. D. Han R. T. Nakao-Inoue H. . (2020). Microglial remodeling of the extracellular matrix promotes synapse plasticity. Cell 182, 388–403.e15. doi: 10.1016/j.cell.2020.05.050, 32615087 PMC7497728

[ref114] Oohashi T. Edamatsu M. Bekku Y. Carulli D. (2015). The hyaluronan and proteoglycan link proteins: organizers of the brain extracellular matrix and key molecules for neuronal function and plasticity. Exp. Neurol. 274, 134–144. doi: 10.1016/j.expneurol.2015.09.010, 26387938

[ref115] Pantazopoulos H. Berretta S. (2016). In sickness and in health: perineuronal nets and synaptic plasticity in psychiatric disorders. Neural Plast. 2016, 1–23. doi: 10.1155/2016/9847696, 26839720 PMC4709762

[ref116] Patel D. C. Swift N. Tewari B. P. Browning J. L. Prim C. Chaunsali L. . (2024). Increased expression of chondroitin sulfate proteoglycans in dentate gyrus and amygdala causes postinfectious seizures. Brain 147, 1856–1870. doi: 10.1093/brain/awad430, 38146224 PMC11068111

[ref117] Perkins K. L. Arranz A. M. Yamaguchi Y. Hrabetova S. (2017). Brain extracellular space, hyaluronan, and the prevention of epileptic seizures. Rev. Neurosci. 28, 869–892. doi: 10.1515/revneuro-2017-0017, 28779572 PMC5705429

[ref119] Pijet B. Stefaniuk M. Kostrzewska-Ksiezyk A. Tsilibary P. E. Tzinia A. Kaczmarek L. (2018). Elevation of MMP-9 levels promotes Epileptogenesis after traumatic brain injury. Mol. Neurobiol. 55, 9294–9306. doi: 10.1007/s12035-018-1061-5, 29667129 PMC6208832

[ref120] Pitkänen A. Engel J. (2014). Past and present definitions of epileptogenesis and its biomarkers. Neurotherapeutics 11, 231–241. doi: 10.1007/s13311-014-0257-2, 24492975 PMC3996117

[ref121] Podgorny O. V. Petukhova E. O. Belousov V. V. (2025). Epilepsy treatment through genetic intervention of GABAergic interneurons: promises and challenges. ACS Chem. Neurosci. 16, 4574–4591. doi: 10.1021/acschemneuro.5c00747, 41332133

[ref122] Pollock E. Everest M. Brown A. Poulter M. O. (2014). Metalloproteinase inhibition prevents inhibitory synapse reorganization and seizure genesis. Neurobiol. Dis. 70, 21–31. doi: 10.1016/j.nbd.2014.06.003, 24946277

[ref123] Pujadas L. Rossi D. Andrés R. Teixeira C. M. Serra-Vidal B. Parcerisas A. . (2014). Reelin delays amyloid-beta fibril formation and rescues cognitive deficits in a model of Alzheimer’s disease. Nat. Commun. 5:3443. doi: 10.1038/ncomms4443, 24599114

[ref124] Rankin-Gee E. K. McRae P. A. Baranov E. Rogers S. Wandrey L. Porter B. E. (2015). Perineuronal net degradation in epilepsy. Epilepsia 56, 1124–1133. doi: 10.1111/epi.13026, 26032766

[ref125] Ray M. H. Williams B. R. Kuppe M. K. Bryant C. D. Logan R. W. (2022). A glitch in the matrix: the role of extracellular matrix remodeling in opioid use disorder. Front. Integr. Neurosci. 16:899637. doi: 10.3389/fnint.2022.899637, 35757099 PMC9218427

[ref126] Reichelt A. C. Hashad A. M. Ghosh-Swaby O. R. Skirzewski M. Scott G. A. Machado M. . (2026). A dual role of hippocampal perineuronal nets in plasticity and protection is revealed by improvement and impairment of diet-induced memory dysfunction. Neuropsychopharmacology 51, 1245–1257. doi: 10.1038/s41386-026-02343-z, 41571987 PMC13212925

[ref127] Riga D. Kramvis I. Koskinen M. K. Van Bokhoven P. Harst J. E. Heistek T. S. . (2017). Hippocampal extracellular matrix alterations contribute to cognitive impairment associated with a chronic depressive-like state in rats. Sci. Transl. Med. 9:eaai8753. doi: 10.1126/scitranslmed.aai8753, 29263233

[ref128] Rogers J. T. Weeber E. J. (2008). Reelin and apoE actions on signal transduction, synaptic function and memory formation. Neuron Glia Biol. 4, 259–270. doi: 10.1017/S1740925X09990184, 19674510

[ref129] Rogers J. T. Zhao L. Trotter J. H. Rusiana I. Peters M. M. Li Q. . (2013). Reelin supplementation recovers sensorimotor gating, synaptic plasticity and associative learning deficits in the heterozygous reeler mouse. J. Psychopharmacol. 27, 386–395. doi: 10.1177/0269881112463468, 23104248 PMC3820099

[ref130] Romberg C. Yang S. Melani R. Andrews M. R. Horner A. E. Spillantini M. G. . (2013). Depletion of perineuronal nets enhances recognition memory and long-term depression in the perirhinal cortex. J. Neurosci. 33, 7057–7065. doi: 10.1523/JNEUROSCI.6267-11.2013, 23595763 PMC3655688

[ref131] Rowlands D. Lensjø K. K. Dinh T. Yang S. Andrews M. R. Hafting T. . (2018). Aggrecan directs extracellular matrix-mediated neuronal plasticity. J. Neurosci. 38, 10102–10113. doi: 10.1523/JNEUROSCI.1122-18.2018, 30282728 PMC6596198

[ref132] Sanchez B. Kraszewski P. Lee S. Cope E. C. (2024). From molecules to behavior: implications for perineuronal net remodeling in learning and memory. J. Neurochem. 168, 1854–1876. doi: 10.1111/jnc.16036, 38158878 PMC12303236

[ref133] Santana J. Marzolo M. P. (2017). The functions of Reelin in membrane trafficking and cytoskeletal dynamics: implications for neuronal migration, polarization and differentiation. Biochem. J. 474, 3137–3165. doi: 10.1042/BCJ20160628, 28887403

[ref134] Scholefield Z. Yates E. A. Wayne G. Amour A. McDowell W. Turnbull J. E. (2003). Heparan sulfate regulates amyloid precursor protein processing by BACE1, the Alzheimer’s beta-secretase. J. Cell Biol. 163, 97–107. doi: 10.1083/jcb.200303059, 14530380 PMC2173449

[ref135] Seiffert E. Dreier J. P. Ivens S. Bechmann I. Tomkins O. Heinemann U. . (2004). Lasting blood-brain barrier disruption induces epileptic focus in the rat somatosensory cortex. J. Neurosci. 24, 7829–7836. doi: 10.1523/JNEUROSCI.1751-04.2004, 15356194 PMC6729929

[ref136] Senkov O. Andjus P. Radenovic L. Soriano E. Dityatev A. (2014). Neural ECM molecules in synaptic plasticity, learning, and memory. Prog. Brain Res. 214, 53–80. doi: 10.1016/B978-0-444-63486-3.00003-7, 25410353

[ref137] Sethi M. K. Zaia J. (2017). Extracellular matrix proteomics in schizophrenia and Alzheimer’s disease. Anal. Bioanal. Chem. 409, 379–394. doi: 10.1007/s00216-016-9900-6, 27601046 PMC5203946

[ref138] Shi W. Wei X. Wang X. du S. Liu W. Song J. . (2019). Perineuronal nets protect long-term memory by limiting activity-dependent inhibition from parvalbumin interneurons. Proc. Natl. Acad. Sci. U. S. A. 116, 27063–27073. doi: 10.1073/pnas.1902680116, 31843906 PMC6936502

[ref139] Siller S. S. Broadie K. (2012). Matrix metalloproteinases and minocycline: therapeutic avenues for fragile X syndrome. Neural Plast. 2012:124548. doi: 10.1155/2012/124548, 22685676 PMC3364018

[ref140] Šimončičová E. Henderson Pekarik K. Vecchiarelli H. A. Lauro C. Maggi L. Tremblay M. È. (2024). Adult neurogenesis, learning and memory. Adv. Neurobiol. 37, 221–242. doi: 10.1007/978-3-031-55529-9_13, 39207695

[ref141] Singh J. B. Perelló-Amorós B. Schneeberg J. Mirzapourdelavar H. Seidenbecher C. I. Fejtová A. . (2026). Activity-dependent extracellular proteolytic cascade cleaves the ECM component brevican to promote structural plasticity. EMBO Rep. 27, 163–185. doi: 10.1038/s44319-025-00644-w, 41261283 PMC12796228

[ref142] Smith A. C. Scofield M. D. Kalivas P. W. (2015). The tetrapartite synapse: extracellular matrix remodeling contributes to corticoaccumbens plasticity underlying drug addiction. Brain Res. 1628, 29–39. doi: 10.1016/j.brainres.2015.03.027, 25838241 PMC4589426

[ref143] Sohal V. S. Zhang F. Yizhar O. Deisseroth K. (2009). Parvalbumin neurons and gamma rhythms enhance cortical circuit performance. Nature 459, 698–702. doi: 10.1038/nature07991, 19396159 PMC3969859

[ref144] Soleman S. Filippov M. A. Dityatev A. Fawcett J. W. (2013). Targeting the neural extracellular matrix in neurological disorders. Neuroscience 253, 194–213. doi: 10.1016/j.neuroscience.2013.08.050, 24012743

[ref145] Sorg B. A. (2012). Reconsolidation of drug memories. Neurosci. Biobehav. Rev. 36, 1400–1417. doi: 10.1016/j.neubiorev.2012.02.004, 22342780 PMC3526670

[ref146] Spijker S. Koskinen M. K. Riga D. (2020). Incubation of depression: ECM assembly and parvalbumin interneurons after stress. Neurosci. Biobehav. Rev. 118, 65–79. doi: 10.1016/j.neubiorev.2020.07.015, 32687884

[ref147] Sterin I. Niazi A. Kim J. Park J. Park S. (2024). Dynamic Organization of Neuronal Extracellular Matrix Revealed by HaloTag-HAPLN1. J. Neurosci. 44:e0666242024. doi: 10.1523/JNEUROSCI.0666-24.2024, 39251350 PMC11502233

[ref148] Stevens S. R. Longley C. M. Ogawa Y. Teliska L. H. Arumanayagam A. S. Nair S. . (2021). Ankyrin-R regulates fast-spiking interneuron excitability through perineuronal nets and Kv3.1b K+ channels. eLife 10:e66491. doi: 10.7554/eLife.66491, 34180393 PMC8257253

[ref149] Stranahan A. M. Erion J. R. Wosiski-Kuhn M. (2013). Reelin signaling in development, maintenance, and plasticity of neural networks. Ageing Res. Rev. 12, 815–822. doi: 10.1016/j.arr.2013.01.005, 23352928 PMC4475282

[ref150] Suttkus A. Holzer M. Morawski M. Arendt T. (2016). The neuronal extracellular matrix restricts distribution and internalization of aggregated tau-protein. Neuroscience 313, 225–235. doi: 10.1016/j.neuroscience.2015.11.040, 26621125

[ref151] Syková E. Nicholson C. (2008). Diffusion in brain extracellular space. Physiol. Rev. 88, 1277–1340. doi: 10.1152/physrev.00027.2007, 18923183 PMC2785730

[ref152] Takács E. Nyilas R. Szepesi Z. Baracskay P. Karlsen B. Røsvold T. . (2010). Matrix metalloproteinase-9 activity increased by two different types of epileptic seizures that do not induce neuronal death: a possible role in homeostatic synaptic plasticity. Neurochem. Int. 56, 799–809. doi: 10.1016/j.neuint.2010.03.003, 20303372

[ref153] Tansley S. Gu N. Guzmán A. U. Cai W. Wong C. Lister K. C. . (2022). Microglia-mediated degradation of perineuronal nets promotes pain. Science 377, 80–86. doi: 10.1126/science.abl6773 35617374

[ref154] Thompson E. H. Lensjø K. K. Wigestrand M. B. Malthe-Sørenssen A. Hafting T. Fyhn M. (2018). Removal of perineuronal nets disrupts recall of a remote fear memory. Proc. Natl. Acad. Sci. U. S. A. 115, 607–612. doi: 10.1073/pnas.1713530115, 29279411 PMC5776974

[ref155] Tsilibary E. Tzinia A. Radenovic L. Stamenkovic V. Lebitko T. Mucha M. . (2014). Neural ECM proteases in learning and synaptic plasticity. Prog. Brain Res. 214, 135–157. doi: 10.1016/B978-0-444-63486-3.00006-2, 25410356

[ref156] Ueno H. Suemitsu S. Murakami S. Kitamura N. Wani K. Okamoto M. . (2017). Postnatal development of GABAergic interneurons and perineuronal nets in mouse temporal cortex subregions. Int. J. Dev. Neurosci. 63, 27–37. doi: 10.1016/j.ijdevneu.2017.08.003, 28859888

[ref157] Vafadari B. Salamian A. Kaczmarek L. (2016). MMP-9 in translation: from molecule to brain physiology, pathology, and therapy. J. Neurochem. 139, 91–114. doi: 10.1111/jnc.13415, 26525923

[ref158] Van Vliet E. A. da Costa Araújo S. Redeker S. Van Schaik R. Aronica E. Gorter J. A. (2007). Blood-brain barrier leakage may lead to progression of temporal lobe epilepsy. Brain 130, 521–534. doi: 10.1093/brain/awl318, 17124188

[ref159] Van’t Spijker H. M. Rowlands D. Rossier J. Haenzi B. Fawcett J. W. Kwok J. C. F. (2019). Neuronal pentraxin 2 binds PNNs and enhances PNN formation. Neural Plast. 2019, 1–13. doi: 10.1155/2019/6804575, 31772567 PMC6854953

[ref160] Wallace B. G. (1989). Agrin-induced specializations contain cytoplasmic, membrane, and extracellular matrix-associated components of the postsynaptic apparatus. J. Neurosci. 9, 1294–1302. doi: 10.1523/JNEUROSCI.09-04-01294.1989, 2539442 PMC6569863

[ref161] Wang X. B. Bozdagi O. Nikitczuk J. S. Zhai Z. W. Zhou Q. Huntley G. W. (2008). Extracellular proteolysis by matrix metalloproteinase-9 drives dendritic spine enlargement and long-term potentiation coordinately. Proc. Natl. Acad. Sci. U. S. A. 105, 19520–19525. doi: 10.1073/pnas.0807248105, 19047646 PMC2614793

[ref162] Wang D. Fawcett J. (2012). The perineuronal net and the control of CNS plasticity. Cell Tissue Res. 349, 147–160. doi: 10.1007/s00441-012-1375-y, 22437874

[ref163] Wareham L. K. Calkins D. J. (2025). Making tracks: microglia and the extracellular matrix. Mol. Neurodegener. 20:101. doi: 10.1186/s13024-025-00898-x, 41024112 PMC12481997

[ref164] Wiera G. Mozrzymas J. W. (2015). Extracellular proteolysis in structural and functional plasticity of mossy fiber synapses in hippocampus. Front. Cell. Neurosci. 9:427. doi: 10.3389/fncel.2015.00427, 26582976 PMC4631828

[ref165] Wiera G. Mozrzymas J. W. (2021). Extracellular metalloproteinases in the plasticity of excitatory and inhibitory synapses. Cells 10:2055. doi: 10.3390/cells10082055, 34440823 PMC8391609

[ref166] Wilczynski G. M. Konopacki F. A. Wilczek E. Lasiecka Z. Gorlewicz A. Michaluk P. . (2008). Important role of matrix metalloproteinase 9 in epileptogenesis. J. Cell Biol. 180, 1021–1035. doi: 10.1083/jcb.200708213, 18332222 PMC2265409

[ref167] Wingert J. C. Sorg B. A. (2021). Impact of Perineuronal nets on electrophysiology of Parvalbumin interneurons, principal neurons, and brain oscillations: a review. Front. Synaptic Neurosci. 13:673210. doi: 10.3389/fnsyn.2021.673210, 34040511 PMC8141737

[ref168] Wlodarczyk J. Mukhina I. Kaczmarek L. Dityatev A. (2011). Extracellular matrix molecules, their receptors, and secreted proteases in synaptic plasticity. Dev. Neurobiol. 71, 1040–1053. doi: 10.1002/dneu.20958, 21793226

[ref169] Wright J. W. Kramár E. A. Meighan S. E. Harding J. W. (2002). Extracellular matrix molecules, long-term potentiation, memory consolidation and the brain angiotensin system. Peptides 23, 221–246. doi: 10.1016/S0196-9781(01)00599-X, 11814638

[ref170] Wu Y. P. Siao C. J. Lu W. Sung T. C. Frohman M. A. Milev P. . (2000). The tissue plasminogen activator (tPA)/plasmin extracellular proteolytic system regulates seizure-induced hippocampal mossy fiber outgrowth through a proteoglycan substrate. J. Cell Biol. 148, 1295–1304. doi: 10.1083/jcb.148.6.1295, 10725341 PMC2174310

[ref171] Xu P. Raanin Chandradoss K. Lukasak B. Paranjapye A. Waldman A. J. Pham K. . (2025). Learning induces persistent chromatin loops underlying robust gene expression during memory recall. bioRxiv 1:8146. doi: 10.1101/2025.09.30.678146

[ref172] Xue Y. X. Xue L. F. Liu J. F. He J. Deng J. H. Sun S. C. . (2014). Depletion of perineuronal nets in the amygdala to enhance the erasure of drug memories. J. Neurosci. 34, 6647–6658. doi: 10.1523/JNEUROSCI.5390-13.2014, 24806690 PMC6608143

[ref173] Yamada J. Jinno S. (2017). Molecular heterogeneity of aggrecan-based perineuronal nets around five subclasses of parvalbumin-expressing neurons in the mouse hippocampus. J. Comp. Neurol. 525, 1234–1249. doi: 10.1002/cne.24132, 27718219

[ref174] Yamaguchi Y. (2000). Lecticans: organizers of the brain extracellular matrix. Cell. Mol. Life Sci. 57, 276–289. doi: 10.1007/PL00000690, 10766023 PMC11146776

[ref175] Yang S. Gigout S. Molinaro A. Naito-Matsui Y. Hilton S. Foscarin S. . (2021). Chondroitin 6-sulphate is required for neuroplasticity and memory in ageing. Mol. Psychiatry 26, 5658–5668. doi: 10.1038/s41380-021-01208-9, 34272488 PMC8758471

[ref176] Yang T. Hu S. Chang W. C. Kao H.-Y. Wang Y. (2022). Perineuronal nets degradation and parvalbumin interneuron loss in a mouse model of DEPDC5-related epilepsy. Dev. Neurosci. 44, 671–677. doi: 10.1159/000525039, 35580549 PMC9669280

[ref177] Yang Q. Yan C. Sun Y. Xie Z. Yang L. Jiang M. . (2024). Extracellular matrix remodeling alleviates memory deficits in Alzheimer’s disease by enhancing the astrocytic autophagy-lysosome pathway. Adv. Sci. 11:e2400480. doi: 10.1002/advs.202400480, 38881515 PMC11336928

[ref178] Yuan W. Matthews R. T. Sandy J. D. Gottschall P. E. (2002). Association between protease-specific proteolytic cleavage of brevican and synaptic loss in the dentate gyrus of kainate-treated rats. Neuroscience 114, 1091–1101. doi: 10.1016/S0306-4522(02)00347-0, 12379262

[ref179] Yutsudo N. Kitagawa H. (2015). Involvement of chondroitin 6-sulfation in temporal lobe epilepsy. Exp. Neurol. 274, 126–133. doi: 10.1016/j.expneurol.2015.07.009, 26231575

[ref180] Zhu K. Fu Y. Zhao Y. Niu B. Lu H. (2025). Perineuronal nets: role in normal brain physiology and aging, and pathology of various diseases. Ageing Res. Rev. 108:102756. doi: 10.1016/j.arr.2025.102756, 40254145

[ref181] Zhu H. X. Li Y. C. Yang X. P. Chu Y.-H. Guo W. Chen R.-X. . (2023). Demographics and clinical characteristics of patients with neurocysticercosis: a retrospective study from Dali, China. Saudi J. Med. Med. Sci. 11, 283–291. doi: 10.4103/sjmms.sjmms_298_23, 37970452 PMC10634465

[ref182] Zhu H. Wang W. Li Y. (2022). Molecular mechanism and regulation of autophagy and its potential role in epilepsy. Cells 11:2621. doi: 10.3390/cells11172621, 36078029 PMC9455075

[ref183] Zhu H. Wang W. Li Y. (2024). The interplay between microbiota and brain-gut axis in epilepsy treatment. Front. Pharmacol. 15:1276551. doi: 10.3389/fphar.2024.1276551, 38344171 PMC10853364

